# Development of the hyolaryngeal architecture in horseshoe bats: insights into the evolution of the pulse generation for laryngeal echolocation

**DOI:** 10.1186/s13227-024-00221-7

**Published:** 2024-02-07

**Authors:** Taro Nojiri, Masaki Takechi, Toshiko Furutera, Nicolas L. M. Brualla, Sachiko Iseki, Dai Fukui, Vuong Tan Tu, Fumiya Meguro, Daisuke Koyabu

**Affiliations:** 1 Graduate School of Medicine, Juntendo University, 2-2-1 Hongo, Bunkyo-Ku, Tokyo 113-8421 Japan; 2 Department of Molecular Craniofacial Embryology, Tokyo Medical and Dental University, 1-5-45 Yushima, Bunkyo-Ku, Tokyo 113-8549 Japan; 3 Department of Infectious Diseases and Public Health, Jockey Club College of Veterinary Medicine and Life Sciences, City University of Hong Kong, Hong Kong SAR, China; 4 The University of Tokyo Fuji Iyashinomori Woodland Study Center, Graduate School of Agricultural and Life Sciences, The University of Tokyo, 341-2 Yamanaka, Yamanakako, Yamanashi 401-05013 Japan; 5 Institute of Ecology and Biological Resources, Vietnam Academy of Science and Technology, No. 18, Hoang Quoc Viet Road, Cau Giay District, Hanoi, Vietnam; 6 Graduate University of Science and Technology, Vietnam Academy of Science and Technology, No. 18, Hoang Quoc Viet Road, Cau Giay District, Hanoi, Vietnam; 7 Research and Development Center for Precision Medicine, University of Tsukuba, 1-2 Kasuga, Tsukuba-Shi, Ibaraki 305-8550 Japan

**Keywords:** Bats, Laryngeal echolocation, Hyolarynx, Trachea, Bioacoustics

## Abstract

**Background:**

The hyolaryngeal apparatus generates biosonar pulses in the laryngeally echolocating bats. The cartilage and muscles comprising the hyolarynx of laryngeally echolocating bats are morphologically modified compared to those of non-bat mammals, as represented by the hypertrophied intrinsic laryngeal muscle. Despite its crucial contribution to laryngeal echolocation, how the development of the hyolarynx in bats differs from that of other mammals is poorly documented. The genus *Rhinolophus* is one of the most sophisticated laryngeal echolocators, with the highest pulse frequency in bats. The present study provides the first detailed description of the three-dimensional anatomy and development of the skeleton, cartilage, muscle, and innervation patterns of the hyolaryngeal apparatus in two species of rhinolophid bats using micro-computed tomography images and serial tissue sections and compares them with those of laboratory mice. Furthermore, we measured the peak frequency of the echolocation pulse in active juvenile and adult individuals to correspond to echolocation pulses with hyolaryngeal morphology at each postnatal stage.

**Results:**

We found that the sagittal crests of the cricoid cartilage separated the dorsal cricoarytenoid muscle in horseshoe bats, indicating that this unique morphology may be required to reinforce the repeated closure movement of the glottis during biosonar pulse emission. We also found that the cricothyroid muscle is ventrally hypertrophied throughout ontogeny, and that the cranial laryngeal nerve has a novel branch supplying the hypertrophied region of this muscle. Our bioacoustic analyses revealed that the peak frequency shows negative allometry against skull growth, and that the volumetric growth of all laryngeal cartilages is correlated with the pulse peak frequency.

**Conclusions:**

The unique patterns of muscle and innervation revealed in this study appear to have been obtained concomitantly with the acquisition of tracheal chambers in rhinolophids and hipposiderids, improving sound intensity during laryngeal echolocation. In addition, significant protrusion of the sagittal crest of the cricoid cartilage and the separated dorsal cricoarytenoid muscle may contribute to the sophisticated biosonar in this laryngeally echolocating lineage. Furthermore, our bioacoustic data suggested that the mineralization of these cartilages underpins the ontogeny of echolocation pulse generation. The results of the present study provide crucial insights into how the anatomy and development of the hyolaryngeal apparatus shape the acoustic diversity in bats.

**Supplementary Information:**

The online version contains supplementary material available at 10.1186/s13227-024-00221-7.

## Background

Vocalization in mammals shows extraordinary acoustic and functional diversity, leading to remarkable diversity in life histories. Among mammals, the mode of sound production varies with feeding strategy [1], habitat [2], and social behavior [3]. The enormous diversity of sound peculiarities such as frequency, intensity, and duration has been attributed to morphological changes in the sound-producing organ, the hyolaryngeal apparatus, as reported in koalas [4], ungulates [5], primates [6–12], manatees [13], rodents [14–18], and bats [19, 20]. Structural simplification of the larynx and laryngeal descent results in vocal complexity in humans [12]. This evidence provides strong support for the idea that variations in mammalian sound signals are shaped by drastic changes in hyolaryngeal anatomy.

Bats, which generate a biosonar sound with vocal folds, laryngeal cartilage, and intrinsic laryngeal muscles for foraging and navigation (laryngeal echolocation), are ideal to explore the link between hyolaryngeal anatomy and bioacoustics. Phylogenetically, extant bats comprise two groups: Yinpterochiroptera (yinpterochiropterans) and Yangochiroptera (yangochiropterans) [21]. Yinpterochiroptera comprises two lineages: Rhinolophoidea (rhinolophoids) and Pteropodidae (pteropodids). Among these, rhinolophoids and yangochiropterans employ laryngeal echolocation [22]. No pteropodids are capable of laryngeal echolocation, but a handful of pteropodid species are known to employ non-laryngeal echolocation using tongue clicks [23] or wing flapping [24]. The yangochiropterans emit biosonar sounds through the oral cavity, except for Phyllostomidae (phyllostomids) and Nycteridae (nycterids), whereas the rhinolophoids, phyllostomids, and nycterids emit biosonar sounds through the nostril by adjusting the acoustic properties of the nose leaf at the snout [25]. Members of yinpterochiropteran families Rhinolophidae (rhinolophids), Hipposideridae (hipposiderids), Rhinopomatidae (rhinopomatids), and yangochiropteran families Nycteridae (nycterids) and Emballonuridae (emballonurids) also possess two pairs of novel sound-modifying organs, known as tracheal chambers, at the lateral and dorsal sides of the tracheal rings [26–28]. This functions as a Helmholtz resonating amplifier to improve sound intensity, possibly contributing to the emission of echolocation pulses through the nostrils [26, 29, 30]. In contrast to other clades that use nasophonation, members of Rhinolophidae and Hipposideridae employ Doppler shift compensation (DSC), which allows them to accurately localize targets by adjusting the sound frequency of ongoing signals to fit them within the most suitable frequency for the primary auditory cortex [31]. For this reason, the rhinolophids and hipposiderids are considered to possess one of the most sophisticated echolocation systems among bats [22].

Previous studies on the evolution of laryngeal echolocation have exclusively focused on auditory organs [32–41]. The cochlea, which transmits sound signals to the cerebral system in vertebrates, is one of the most iconic organs because it is markedly enlarged in laryngeally echolocating bats [32]. In addition, laryngeally echolocating bats possess a fully ossified stylohyal attached to the ectotympanic bone, which improves their sensitivity to ongoing signals [34, 42]. The morphology and development of these features have been well-documented in the context of the evolutionary origins of laryngeal echolocation in bats [31, 32, 35–38, 41, 42]. The enlarged inner ear and ectotympanic-stylohyal unit develop heterotopically and heterochronically in rhinolophoids and yangochiropterans, supporting the multiple-origin hypothesis of laryngeal echolocation in bats [39]. The auditory organs for perceiving echolocation pulses have undergone independent developmental changes, presumably reflecting the differences in biosonar pulse emissions among bats.

Concerning the sound-producing organs of bats, a few studies have reported the musculoskeletal morphology of the hyolaryngeal apparatus in adults [26, 40, 43–49]. Laryngeally echolocating bats possess hypertrophied intrinsic laryngeal muscles supported by reinforced cricoid, thyroid, and arytenoid cartilage [26, 44, 50–52]. In particular, the cricothyroid muscle is a superfast muscle essential for the generation of high-frequency sounds in yangochiropterans [19]. Given that the laryngeal cartilage associated with these muscles is gradually mineralized after birth [28, 46, 48], it is predicted that the ontogeny of biosonar pulse emission is linked to laryngeal growth [47]. However, the paucity of our knowledge on the development of the entire anatomy of the hyolaryngeal apparatus and the acoustic peculiarities of the echolocation pulse restrict our understanding of how morphological changes in the vocal apparatus shape the echolocation pulse during ontogeny.

The laryngeal cartilages serve as the support organ between the hyoid and trachea, anchoring the intrinsic laryngeal muscles essential for vocalization. Previous study on laboratory mice revealed that the size of the cricoid, thyroid, arytenoid, and epiglottic cartilages have been shown to significantly vary with mean fundamental frequency [15]. In the case of the laryngeally echolocating bats, furthermore, due to the mechanical stress by the superfast muscle contraction, the cricoid cartilage of the phyllostomids is calcified in the postnatal individual with immature flight and laryngeal echolocation [48]. Given these facts, it is expected that postnatal calcification of the laryngeal cartilages would improve the sound frequency in mammals. It should be noted that rats and mice also employ the ultrasonic vocalizations (USVs) [53], but unlike laryngeally echolocating bats, they do not possess the superfast muscle in their larynx. Thus, the postnatal calcification dynamics of the laryngeal cartilages are predicted to be qualitatively deviated in laryngeally echolocating bats against mice.

Here, we conducted the first detailed description of the pre- and postnatal development of the hyolaryngeal apparatus and compared morphological development and pulse ontogeny in horseshoe bats (the genus *Rhinolophus*). The hyolarynx was visualized three-dimensionally using microcomputed tomography (micro-CT). In addition, using a diffusible iodine-based contrast-enhanced micro-CT technique (DiceCT) [54, 55], we described the morphology of laryngeal cartilage and intrinsic laryngeal muscles in detail for the first time in bats. Using immunohistochemistry, we described the three-dimensional morphology of the cartilage, muscles, and the innervation patterns of the hyolaryngeal complex. To validate the anatomical peculiarities of bats against other mammals, we compared the hyolaryngeal development of horseshoe bats with that of laboratory mice (*Mus musculus*). Due to the similar body size to the horseshoe bats examined here and previous broad applications for studying laryngeal maturation and disorder [56], the laboratory mouse is an ideal outgroup for the comparative embryology of the hyolaryngeal apparatus. Furthermore, we measured the maximum frequency of each harmonic pulse, CF_1_, and CF_2_, from postnatal day 0 to adult individuals and compared pulse ontogeny with morphological development. Our study highlights the unique anatomical and developmental patterns of the laryngeal cartilage, cricothyroid muscle, and cranial laryngeal nerve in laryngeally echolocating bats, providing tangible insights into how the anatomy and development of the hyolarynx shape the acoustic diversity in bats.

## Results

### General morphology of the hyolaryngeal complex in horseshoe bats

#### Hyoid

**Fig. 1:**
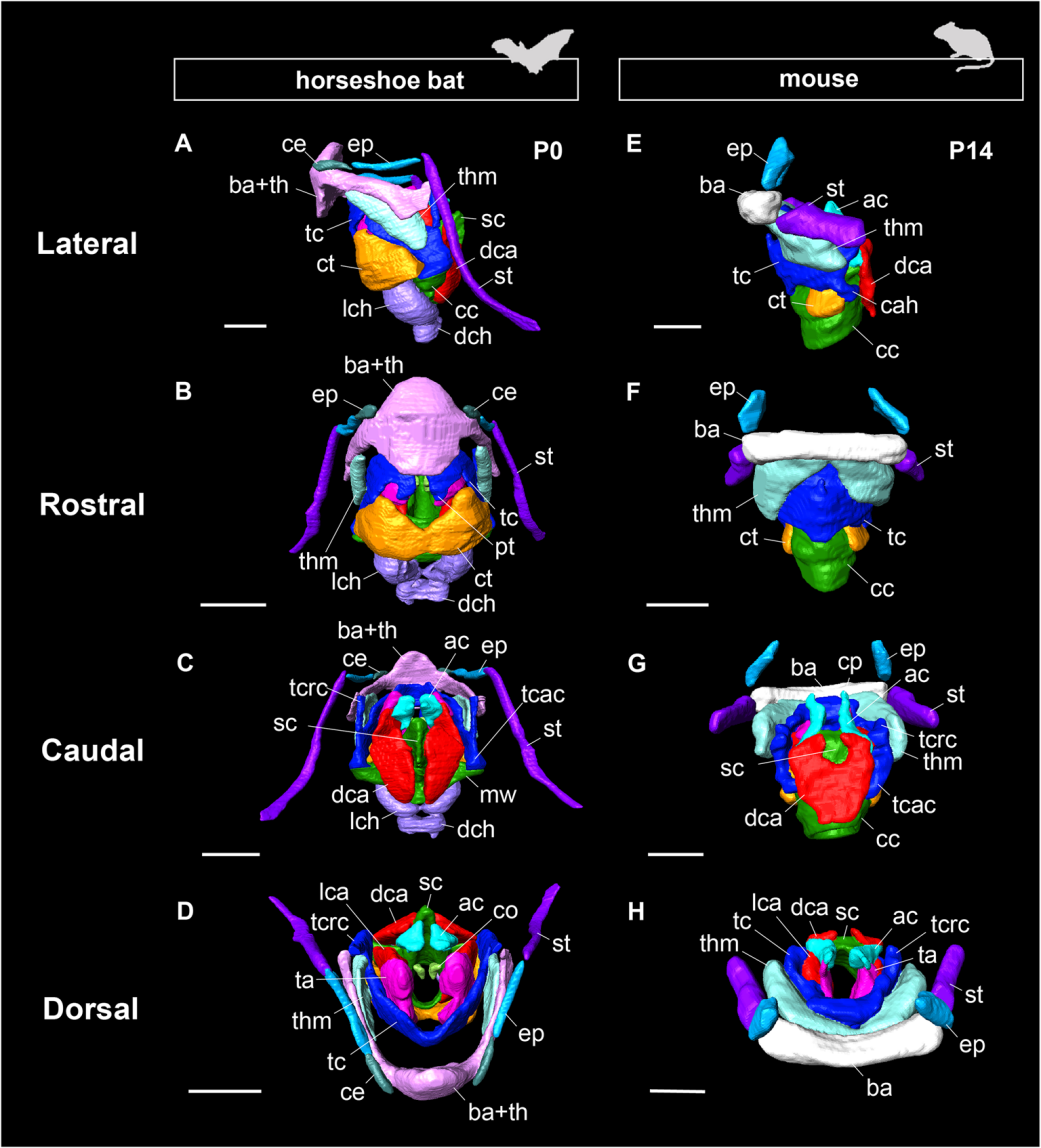
The gross anatomy of the hyolaryngeal apparatus in bats (postnatal day 0 of *Rhinolophus pusillus*) and mice (postnatal day 14 of *Mus musculus*) reconstructed with the diffusible iodine-based contrast-enhanced CT scanning. Left side of the hyolaryngeal morphology is shown in lateral view. Scale bars = 1 mm. See text for abbreviations

The hyoid apparatus of horseshoe bats consists of five bony elements: basihyal, thyrohyal, ceratohyal, epihyal, and stylohyal, whereas in mice, it includes only three elements: basihyal, epihyal, and stylohyal (Fig. 1). In horseshoe bats, the basihyal and thyrohyal bones were fully fused, and the boundary was not evident (Fig. 1A). The epihyal and stylohyal bones were proportionally elongated compared with those in mice. There was a large gap between the basihyal bone and thyroid cartilage, unlike in mice (Fig. 1D, H). The thyrohyoid bone of mice is connected to the thyroid cartilage only by the thyrohyoid muscle attached to the ventral side of the basihyal and rostrolateral portion of the thyroid cartilage; however, in horseshoe bats, the thyrohyoid muscle and caudal tip of the thyrohyal bone were connected to the lateral side and thyroid cranial cornua, respectively (Fig. 1A).

#### Larynx

**Fig. 2:**
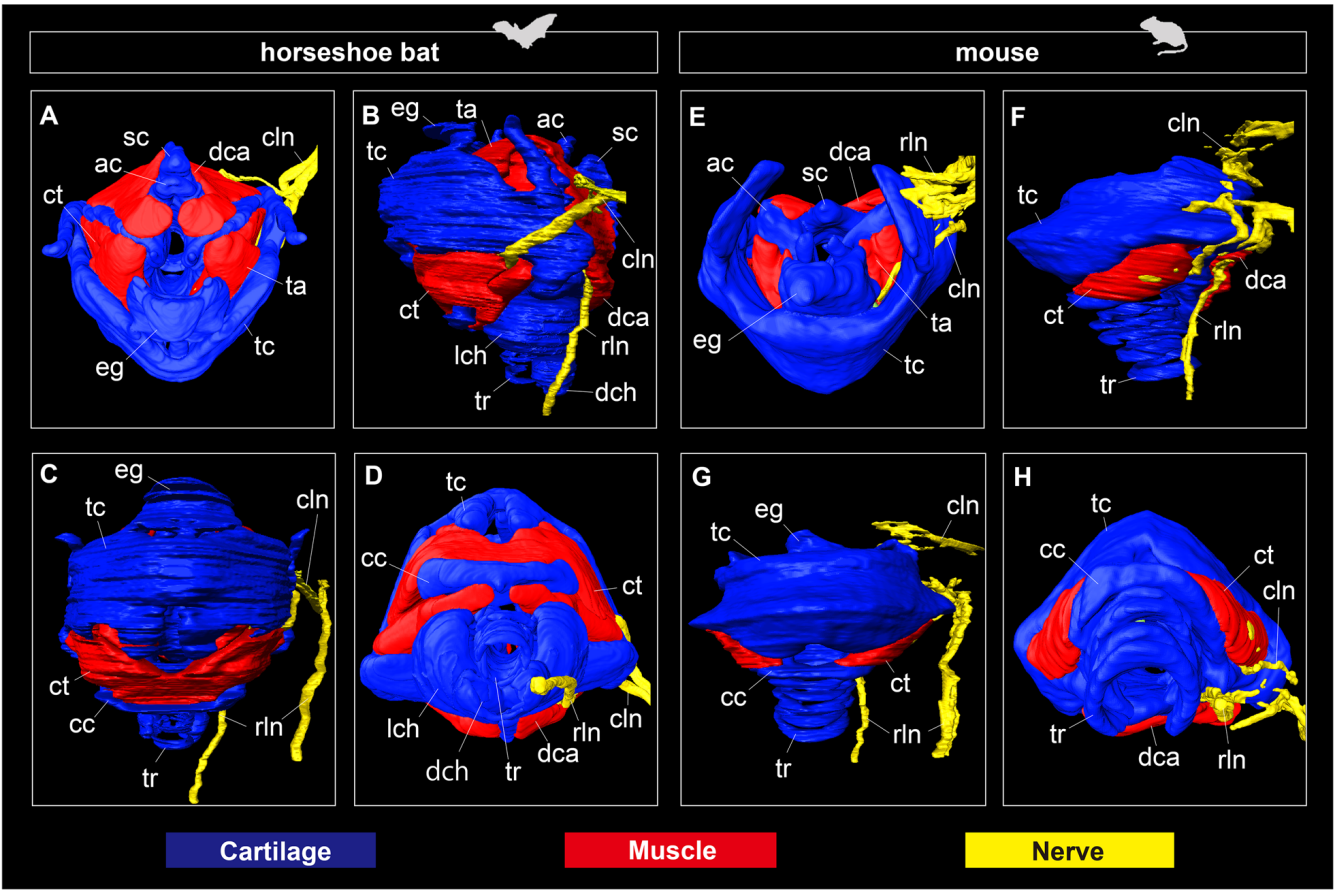
The anatomical module of the larynx in CS22 individual of the horseshoe bat (*Rhinolophus malayanus*) and E18.5 individual of the laboratory mouse (*Mus musculus*). Green color refers to the cartilage: epiglottic cartilage, thyroid cartilage, cricoid cartilage, arytenoid cartilage, lateral and dorsal tracheal chamber, and tracheal rings. Red color refers to the muscle: cricothyroid muscle, dorsal and lateral cricoarytenoid muscle, and thyroarytenoid muscle. Blue color refers to the nerve: cranial and recurrent laryngeal nerve. **A** Dorsal view of the larynx of CS22 of *R. malayanus*. **B** Lateral view of the larynx of CS22 of *R. malayanus*. **C** Rostral view of the larynx of CS22 of *R. malayanus*. **D** Ventral view of the larynx of CS22 of *R. malayanus*. **E** Dorsal view of the larynx of E18.5 of *M. musculus*. **F** Lateral view of the larynx of E18.5 of *M. musculus*. **G** Rostral view of the larynx of E18.5 of *M. musculus*. **H** Ventral view of the larynx of E18.5 of *M. musculus*

Each anatomical module (cartilage, muscle, and nerve) is illustrated in Fig. 2. The cricoid cartilage had a sagittal crest in horseshoe bats, completely dividing the dorsal cricoarytenoid muscle into left and right components (Figs. 1A–C, 2A). The thyroid cartilage had the cranial and caudal cornua connected to the thyrohyal bone and muscular wings of the cricoid cartilage in bats. The rostroventral tip of the thyroid cartilage was separated to form a pair of protuberances, to which the thyroarytenoid muscle was attached (Fig. 1B). While the thyroid cartilage of mice had one pair of the foramen thyroideum at the lamina of the thyroid cartilage, through which the cranial laryngeal nerve passes, that of the thyroid cartilage was not opened in the rhinolophoids. The cricothyroid muscle surrounding the cranial arch of the cricoid cartilage was significantly enlarged in bats compared to that in mice. While the cricothyroid muscle was separated into left and right components in mice, those of horseshoe bats met at the median line, forming one large muscle complex.

#### Trachea

A pair of lateral and dorsal tracheal chambers were present in the ventral portion of the cranial arch of the cricoid cartilage (Figs. 1A–D, 2A–D). The lateral tracheal chamber was completely attached to the ventral side of the cricoid cartilage and the dorsal tracheal chamber. Both tracheal chambers had a pouch-shaped structure surrounded by the glottis and tracheal rings and had internal cavities connected to the respiratory tract. The ventral portion of the cricothyroid muscle was attached to the dorsal side of the lateral tracheal chamber, denoting that the contraction of the tracheal chamber was regulated by the common muscle to generate the biosonar pulse.

#### Innervation

**Fig. 3:**
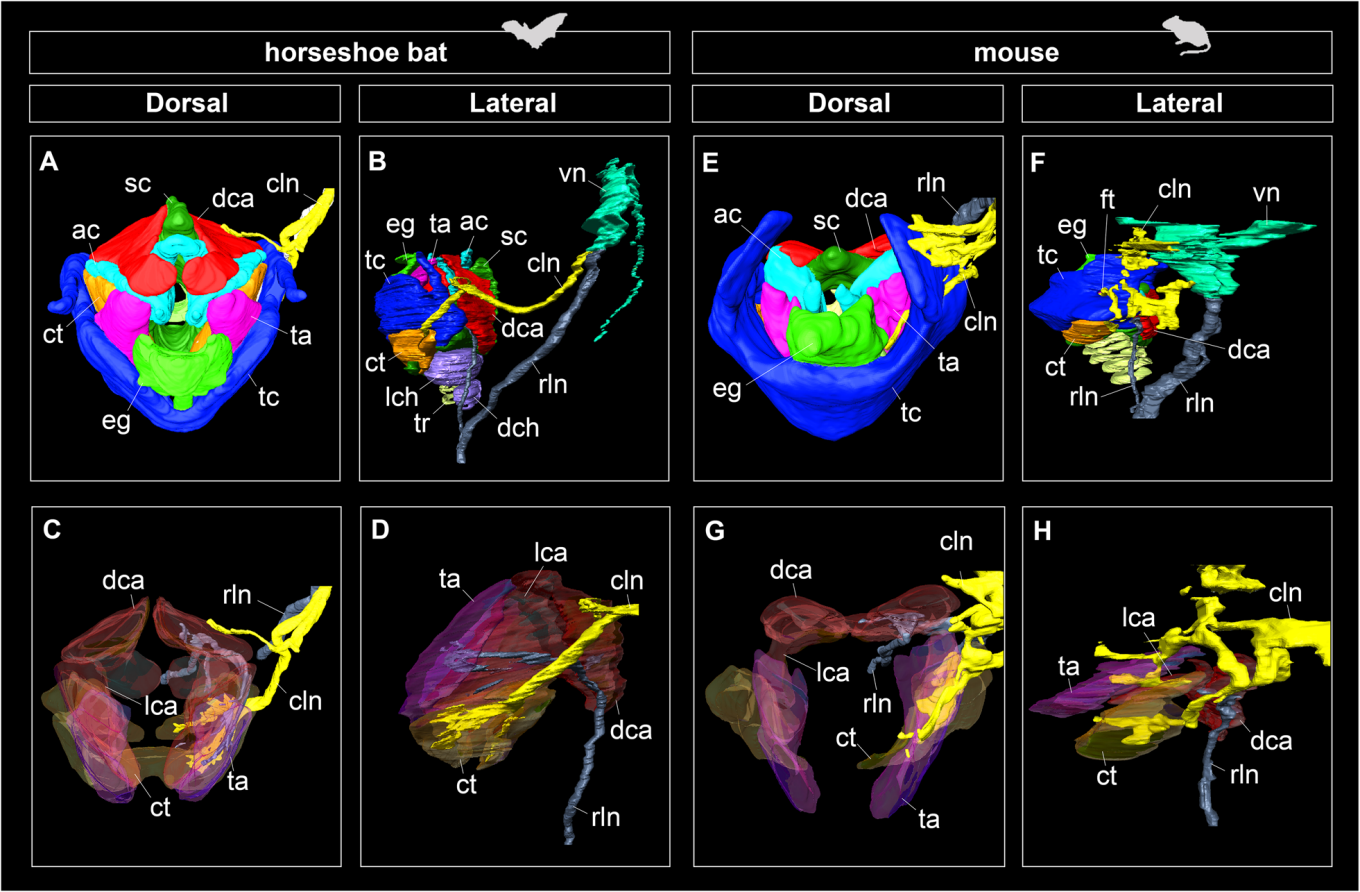
The whole morphology of the intrinsic laryngeal muscle and innervations of the cranial laryngeal nerve and recurrent laryngeal nerve of CS22 stage in *Rhinolophus malayanus* and E18.5 stage in *Mus musculus*. **A** Dorsal view of the hyolaryngeal cartilages, intrinsic laryngeal muscles, and vagus nerve of CS22 fetus of *R. malayanus*. **B** Lateral view of the hyolaryngeal cartilages, intrinsic laryngeal muscles, and vagus nerve of CS 22 fetus of *R. malayanus*. **C** Dorsal view of the intrinsic laryngeal muscles and vagus nerve of CS22 fetus of *R. malayanus*. **D** Lateral view of the intrinsic laryngeal muscles and vagus nerve of CS22 fetus of *R. malayanus*. **E** Dorsal view of the hyolaryngeal cartilages, intrinsic laryngeal muscles, cranial laryngeal nerve, and recurrent laryngeal nerve of E18.5 fetus of *M. musculus*. **F** Lateral view of the hyolaryngeal cartilages, intrinsic laryngeal muscles, cranial laryngeal nerve, and recurrent laryngeal nerve of E18.5 fetus of *M. musculus*. **G** Dorsal view of the intrinsic laryngeal muscles, cranial laryngeal nerve, and recurrent laryngeal nerve of E18.5 fetus of *M. musculus*. **H** Lateral view of the intrinsic laryngeal muscles, cranial laryngeal nerve, and recurrent laryngeal nerve of E18.5 fetus of *M. musculus*. The cranial laryngeal nerve and recurrent laryngeal nerve were visualized using the immunohistochemistry of the acetylated tubulin antibody. The left side of the hyolaryngeal morphology is shown in lateral view. See text for abbreviations

Figures 2 and 3 show the innervation pattern of the laryngeal muscles in CS22 of the horseshoe bat and E18.5 of the laboratory mouse, which were three-dimensionally reconstructed using immunohistochemical staining. The vagus nerve (cranial nerve X) had two branches, the cranial and recurrent laryngeal nerves. In mice, the cranial laryngeal nerve passed through the foramen thyroideum (Figs. 2E–H, 3E, F), whereas in bats, it passed below the thyroid cartilage and supplied the cricothyroid muscle (Figs. 2A–D, 3B). In the cricothyroid muscle of bats, the cranial laryngeal nerve had two branches, rostral and ventral (Fig. 3D). These two branches passed through the rostral and ventral cricothyroid muscles, indicating that the ventral branch of the cranial laryngeal nerve supplied contractions to the lateral tracheal chamber. The recurrent laryngeal nerve, previously supplied in the postcranial direction, looped under the aortic arch, and reached the dorsal cricoarytenoid muscle in both bats and mice (Fig. 3D, H). The recurrent laryngeal nerve passed the dorsal cricoarytenoid muscle, was supplied to the lateral cricoarytenoid muscle, and finally to the thyroarytenoid muscle (Fig. 3C, G). The supply pattern of the recurrent laryngeal nerve was common between bats and mice.

### Prenatal development of the hyolaryngeal complex in horseshoe bats and in mice

#### Hyoid development

**Fig. 4:**
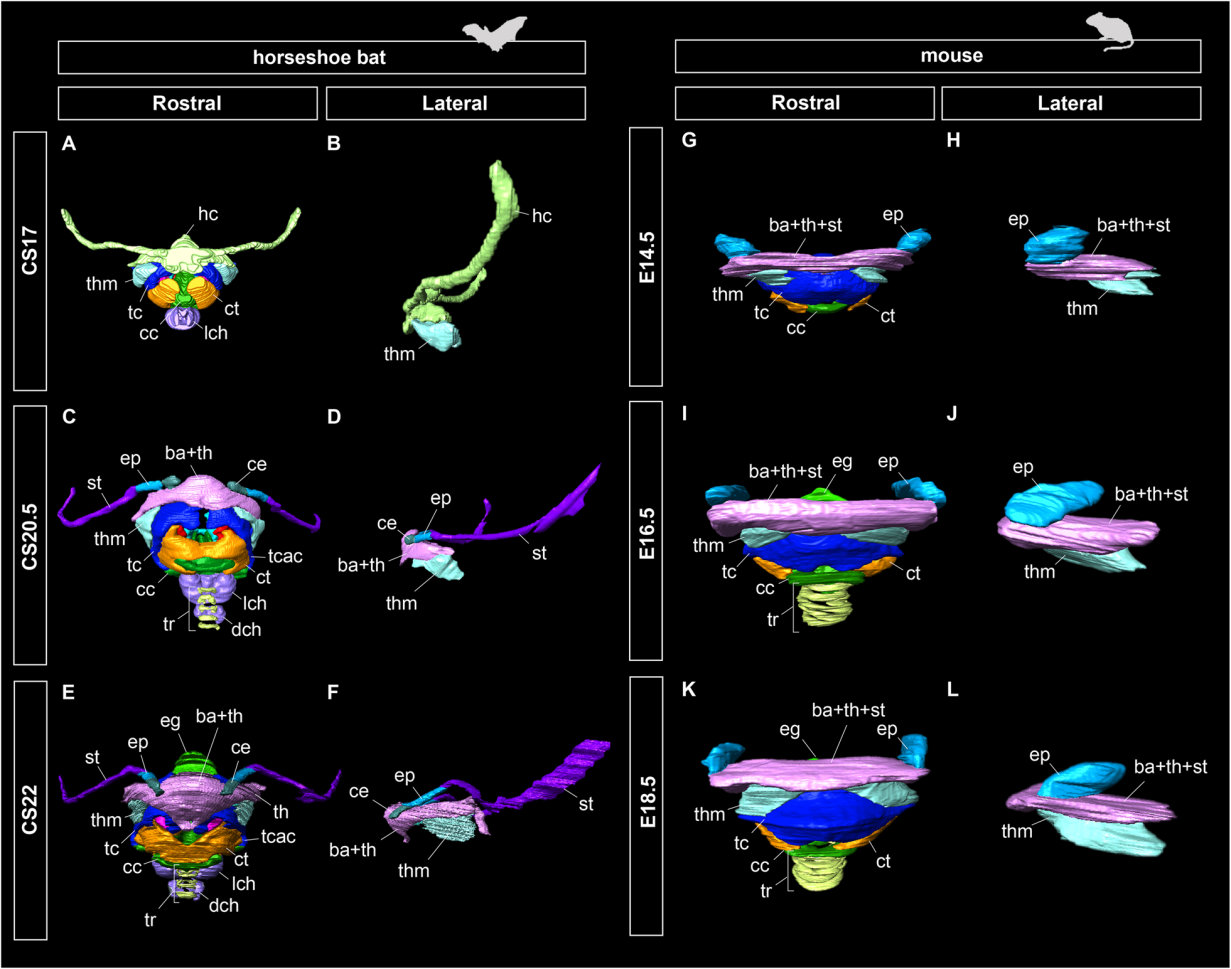
Prenatal development of the hyoid apparatus in horseshoe bats (CS17, CS20.5, CS22) and *Mus musculus* (E14.5, E16.5, E18.5), three-dimensionally reconstructed using serial tissue sections. **A** Rostral view of the hyolaryngeal apparatus of CS17 fetus of the horseshoe bat. **B** Lateral view of the hyoid components of CS17 fetus of *Rhinolophus pusillus*. **C** Rostral view of the hyolaryngeal apparatus of CS20.5 fetus of *R. malayanus*. **D** Lateral view of the hyoid components of CS20.5 fetus of *R. malayanus*. **E** Rostral view of the hyolaryngeal apparatus of CS22 fetus of *R. malayanus*. **F** Lateral view of the hyoid components of CS22 fetus of *R. malayanus*. **G** Rostral view of the hyolaryngeal apparatus of E14.5 fetus of *M. musculus*. **H** Lateral view of the hyoid components of E14.5 fetus of *M. musculus*. **I** Rostral view of the hyolaryngeal apparatus of E16.5 fetus of *M. musculus*. **J** Lateral view of the hyoid components of E16.5 fetus of *M. musculus*. **K** Rostral view of the hyolaryngeal apparatus of E18.5 fetus of *M. musculus*. **L** Lateral view of the hyoid components of E18.5 fetus of *M. musculus*. The tracheal chambers and tracheal rings of the horseshoe bats start those chondrifications at CS17 and CS20.5, respectively. The left side of the hyolaryngeal morphology is shown in lateral view. See text for abbreviations

Cartilaginous condensation began at mid-fetal stage (CS17) (Fig. 4A, B) in *Rhinolophus* bats. The cranial cornua, consisting of the ceratohyal, epihyal, and stylohyal, was separated from the hyoid body (basihyal) and caudal cornua (thyrohyal), but was not segmented to form each component. The thyrohyoid muscle, connecting the hyoid apparatus to the laryngeal cartilage, was present in the rostrolateral portion of the hyoid complex and thyroid cartilage.

The condensed hyoid chondrocytes differentiated into ceratohyal, epihyal, and stylohyal at late-fetal stage (CS20.5) (Fig. 4C, D). Periosteum was observed around the stylohyal bone, denoting that the ossification started at this stage. The general morphology of the hyoid cartilage and muscle did not change from CS20.5 onward (Fig. 4E, F).

In *Mus musculus*, the epihyal, basihyal, thyrohyal, stylohyal cartilages, and thyrohyoid muscles were already evident at day 14.5 (E14.5), but the boundaries in each hyoid component were not evident (Fig. 4G, H). A connection between the hyoid and larynx was also achieved at this stage, after which there was no drastic change in the overall morphology of the hyolaryngeal complex (Fig. 4K, L).

#### Laryngeal development

**Fig. 5:**
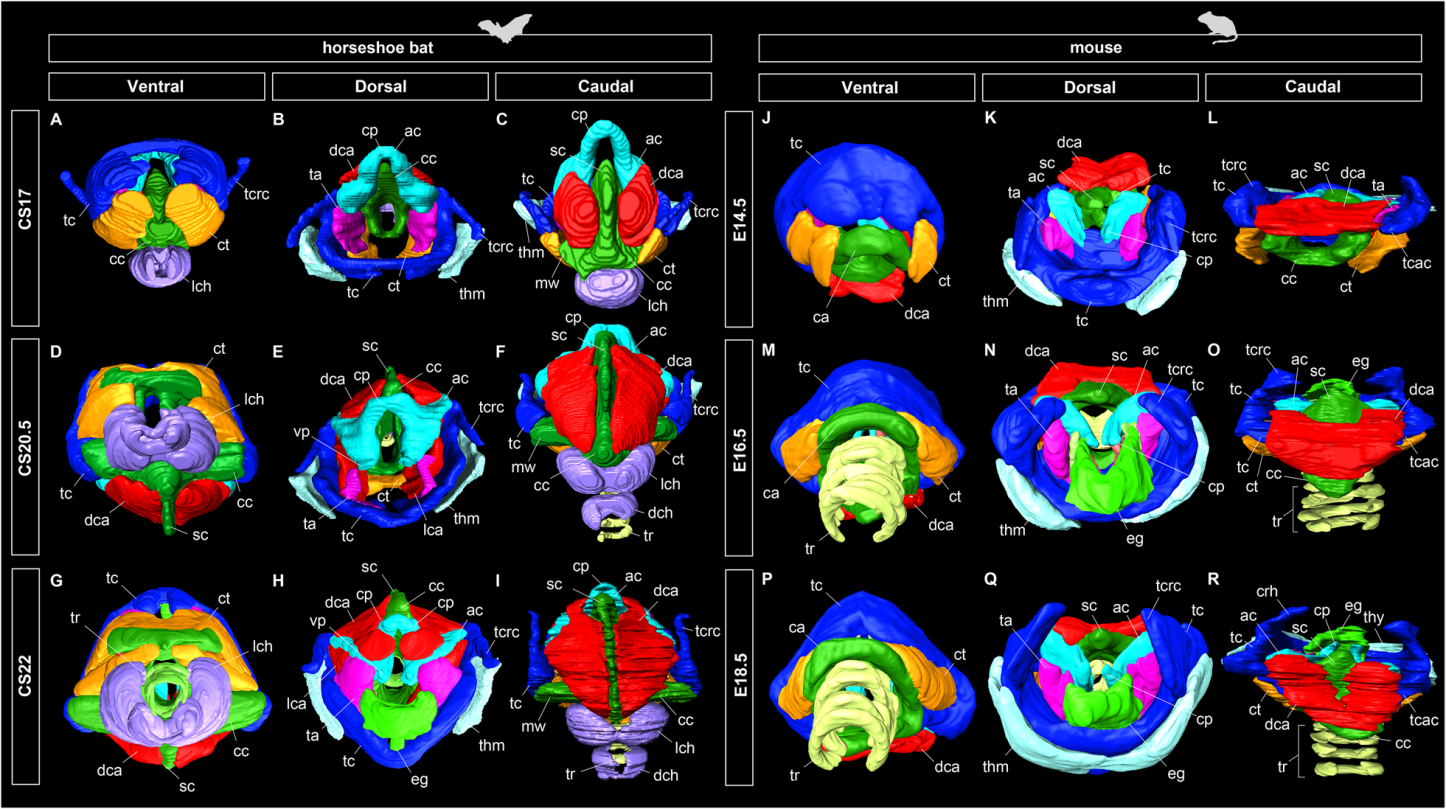
Prenatal laryngeal development of horseshoe bats and laboratory mice, three-dimensionally reconstructed from serial tissue sections. See text for abbreviations. **A** Ventral view of the hyolaryngeal apparatus of CS17 fetus of *Rhinolophus pusillus*. **B** Dorsal view of the hyolaryngeal apparatus of CS17 fetus of *R. pusillus*. **C** Caudal view of the hyolaryngeal apparatus of CS17 fetus of *R. pusillus*. **D** Ventral view of the hyolaryngeal apparatus of CS20.5 fetus of *R. pusillus*. **E** Dorsal view of the hyolaryngeal apparatus of CS20.5 fetus of *R. malayanus*. **F** Caudal view of the hyolaryngeal apparatus of CS20.5 fetus of *R. malayanus*. **G** Ventral view of the hyolaryngeal apparatus of CS22 fetus of *R. pusillus*. **H** Dorsal view of the hyolaryngeal apparatus of CS22 fetus of *R. malayanus*. **I** Caudal view of the hyolaryngeal apparatus of CS22 fetus of *R. malayanus*. **J** Ventral view of the hyolaryngeal apparatus of E14.5 fetus of *Mus musculus*. **K** Dorsal view of the hyolaryngeal apparatus of E14.5 fetus of *M. musculus*. **L** Caudal view of the hyolaryngeal apparatus of E14.5 fetus of *M. musculus*. **M** Ventral view of the hyolaryngeal apparatus of E16.5 fetus of *M. musculus*. **N** Dorsal view of the hyolaryngeal apparatus of E16.5 fetus of *M. musculus*. **O** Caudal view of the hyolaryngeal apparatus of E16.5 fetus of *M. musculus*. **P** Ventral view of the hyolaryngeal apparatus of E18.5 fetus of *M. musculus*. **Q** Dorsal view of the hyolaryngeal apparatus of E18.5 fetus of *M. musculus*. **R** Caudal view of the hyolaryngeal apparatus of E18.5 fetus of *M. musculus*

Chondrification of the cricoid, thyroid, and arytenoid began at mid-fetal stage (CS17) in horseshoe bats. The cranial arch was formed at the ventral margin of the cricoid cartilage that surrounds the glottis. The sagittal crest projected dorsally from the medial portion of the cricoid cartilage. The cranial cornua of the thyroid cartilage was dorsally projected to connect to the hyoid complex. The corniculate processes of the arytenoid cartilage were by now in contact with one another. The cricothyroid, cricoarytenoid, and thyroarytenoid muscles were present in CS17 (Fig. 5A–C). The cricothyroid muscle was formed at the rostroventral portion of the thyroid cartilage and the rostrodorsal portion of the cricoid cartilage, covering the cranial arch of the cricoid cartilage. The thyroarytenoid muscle was formed at the ventral part of the arytenoid cartilage, protruding toward the thyroid cartilage. The lateral and dorsal cricoarytenoid muscles were formed at the rostral portion of the arytenoid cartilage and the caudal surface of the cricoid cartilage, respectively. The dorsal cricoarytenoid muscle was separated into left and right components by the sagittal crest of the cricoid cartilage (Fig. 5C).

The muscular wings of the cricoid cartilage projected bilaterally to form a joint with the caudal cornua of the thyroid cartilage in late fetal stage (CS20.5) (Fig. 5E, F). The sagittal crest of the cricoid cartilage was projected to be greater than that of the CS17. The dorsal tip of the cranial cornua of the thyroid cartilage was slightly curved. In dorsal view, the rostral tip of the thyroid cartilage was curved, forming a V-shaped morphology (Fig. 5E). The vocal processes of the arytenoid cartilage were projected medially around the glottis. The muscular process of the arytenoid cartilage also projected bilaterally. The cricothyroid muscle further expanded toward the tracheal region to surround the cranial arch of the cricoid cartilage. The thyroarytenoid muscle extended rostrally and attached to thyroid cartilage. The dorsal cricoarytenoid muscle expanded dorsoventrally along the sagittal crest of cricoid cartilage.

At later fetal stage (CS22), the caudal cornua of the thyroid cartilage and muscular wings of the cricoid cartilage projected in the ventral and bilateral directions, respectively. The projection of the front portion occurred in the rostrolateral portion of the cranial arch of the cricoid cartilage. The rostral tip of the thyroid cartilage became more convex than in the previous stage. The muscular processes of the arytenoid cartilage protruded bilaterally to expand the attachment area of the dorsal cricoarytenoid muscle. The cricothyroid muscle expanded medially and the cranial arch of the cricoid cartilage was fully covered. The lateral portion of the cricothyroid muscle extended beyond the front plate of the cricoid cartilage and reached the trachea. The thyroarytenoid muscle extended rostrolaterally to expand the area attached to the thyroid cartilage. The dorsal cricoarytenoid muscle extended dorsorostrally and was attached to the depression between the corniculate and the vocal processes of the arytenoid cartilage.

Similarly, in mice, the cricoid, thyroid, and arytenoid cartilages were already chondrified at E14.5 (Fig. 5J). The sagittal crest of the cricoid cartilage was slightly projected. The cranial arch of the cricoid cartilage surrounding the glottis was evident. The thyroid cranial cornua projected dorsally to form a joint with the hyoid complex. The rostral tip of the thyroid cartilage was convex in the dorsal view. The arytenoid cartilage had already undergone a corniculate process. The cricothyroid muscle was also evident, running between the ventrocaudal and dorsorostral portions of the thyroid cartilage. The thyroarytenoid muscle was formed at the rostral site of the arytenoid cartilage and attached to the caudal side of the thyroid cartilage. The lateral cricoarytenoid muscle at the rostroventral site of the arytenoid cartilage remained small. The dorsal cricoarytenoid muscle was still not in contact with the lateral cricoarytenoid muscle and was not separated by the sagittal crest of the cricoid cartilage, similar to that in horseshoe bats (Fig. 5L). At E16.5, the cricoid cartilage expanded ventrally to expand the attachment area of the dorsal cricoarytenoid muscle. The thyroid cartilage was in contact with the muscular wings of the cricoid cartilage. In contrast to horseshoe bats, a pair of the foramen thyroideum was opened at the lateral lamina of the thyroid cartilage, through which the cranial laryngeal nerve passes. From E16.5 onward, the morphology of the laryngeal cartilage did not change drastically (Fig. 5M–O). At E18.5, the dorsal cricoarytenoid muscle was separated by the caudally projected sagittal crest of the cricoid cartilage but was not fully divided, as in bats (Fig. 5P–R). As for the cricothyroid muscle, the rostral tips of the left and right muscles did not meet or reach the trachea; thus, the cranial arch of the cricoid cartilage remained exposed, unlike in horseshoe bats.

#### Tracheal development

**Fig. 6:**
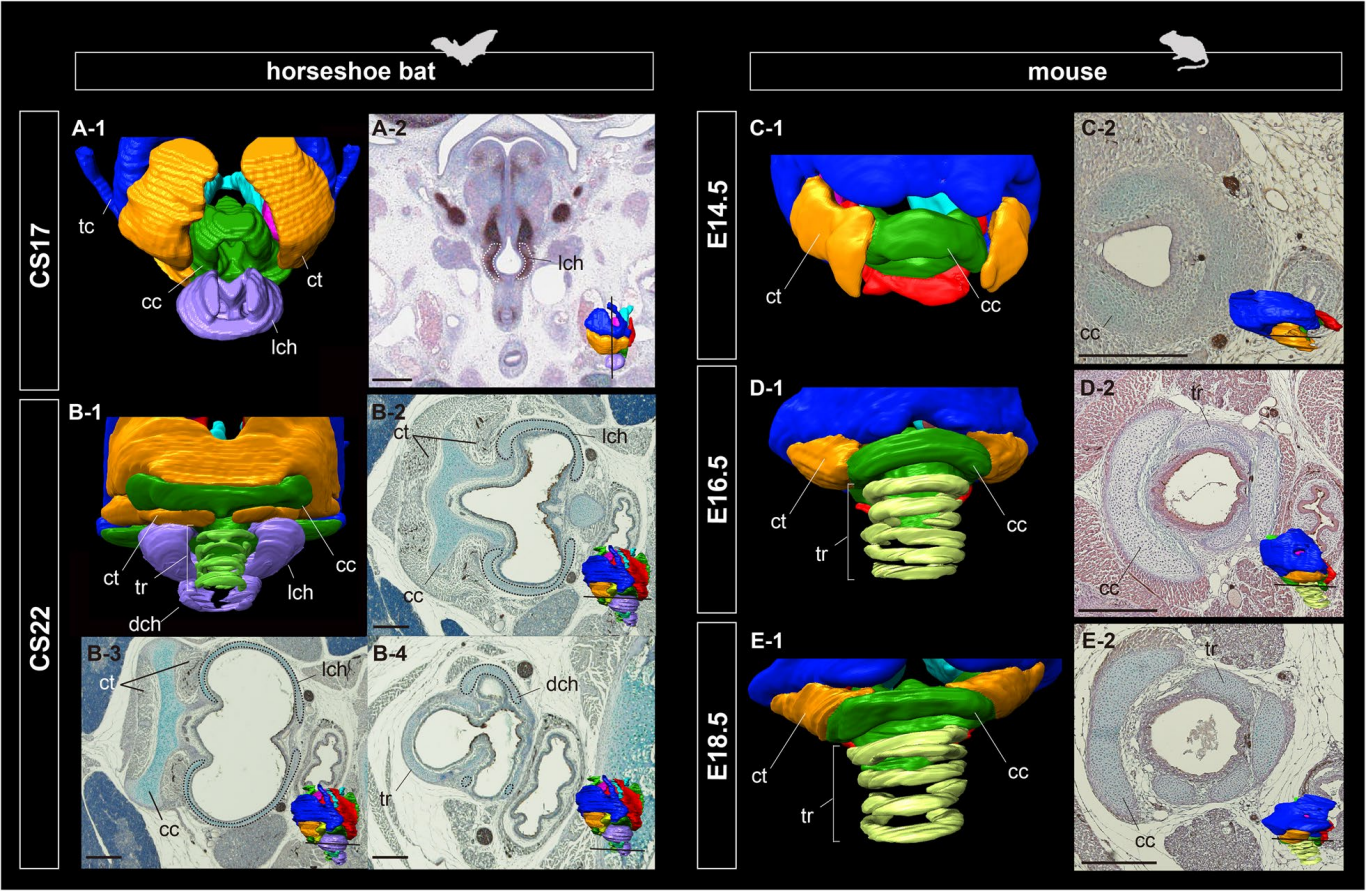
The three-dimensional reconstruction of the fetal tracheal development and histological observations in horseshoe bats and laboratory mice using immunohistochemistry. **A-1** The tracheal anatomy of CS17 fetal specimen of *Rhinolophus pusillus*. **A-2** The histological section of the hyolarynx of CS17 fetal specimen of *R. pusillus*, stained by hematoxylin and immunohistochemistry of Sox9. **B-1** The tracheal anatomy of CS22 fetal specimen of *R. malayanus*. **B-2** to **B-4** The histological sections of the hyolarynx of CS22 fetal specimen of *R. malayanus*, stained by alcian blue, hematoxylin, and immunohistochemistry of acetylated tubulin antibody. **C-1** The tracheal anatomy of E14.5 fetal specimen of *Mus musculus*. **C-2** The histological section of the hyolarynx of E14.5 fetal specimen of *M. musculus*, stained by alcian blue, hematoxylin, and immunohistochemistry of acetylated tubulin antibody. **D-1** The tracheal anatomy of E16.5 fetal specimen of *M. musculus*. **D-2** the histological section of the hyolarynx of E16.5 fetal specimen of *M. musculus*, stained by alcian blue, hematoxylin, and immunohistochemistry of acetylated tubulin antibody. **E-1** The tracheal anatomy of E18.5 fetal specimen of *M. musculus*. **E-2** The histological section of the hyolarynx of E18.5 fetal specimen of *M. musculus*, stained by alcian blue, hematoxylin, and immunohistochemistry of acetylated tubulin antibody. Scale bars = 500 μm. See text for abbreviations

In horseshoe bats, chondrification of the tracheal ring was not evident at mid-fetal stage (CS17), whereas chondrocytes were condensed in the region of the lateral tracheal chamber, denoting that the lateral tracheal chamber is a novel laryngeal cartilage acquired in horseshoe bats (Fig. 6A). Condensed chondrocytes in the lateral tracheal chamber were continuous with those in the cricoid cartilage (Fig. 6A-2). At late-fetal stage (CS22), the tracheal rings began to chondrify (Fig. 6B). Simultaneously, the dorsal tracheal chamber was chondrified ventrally into the lateral tracheal chamber. The condensed chondrocytes in both the lateral and dorsal tracheal chambers were separated to form the left and right tracheal chambers (Fig. 6B-2, B-3). The lateral tracheal chamber covered the tracheal region from the cranial arch of the cricoid cartilage to the third tracheal ring, while the dorsal tracheal chamber covered the fourth to sixth tracheal rings. The lateral tracheal chamber was articulated with the cranial arch of the cricoid cartilage (Fig. 6B-2), whereas the dorsal tracheal chamber was connected to the sixth tracheal ring (Fig. 6B-4). The ventral portion of the cricothyroid muscle reached the caudal side of a pair of lateral tracheal chambers.

In laboratory mice, tracheal rings were not evident at E14.5 with all components of the laryngeal cartilage (Fig. 6C). Tracheal rings were chondrified ventrally to the cranial arch of the cricoid cartilage at E16.5 (Fig. 6D), after which there was no drastic shift in the morphology of the tracheal region (Fig. 6E).

### Pre- and postnatal ossification and mineralization of the hyolaryngeal apparatus

**Fig. 7:**
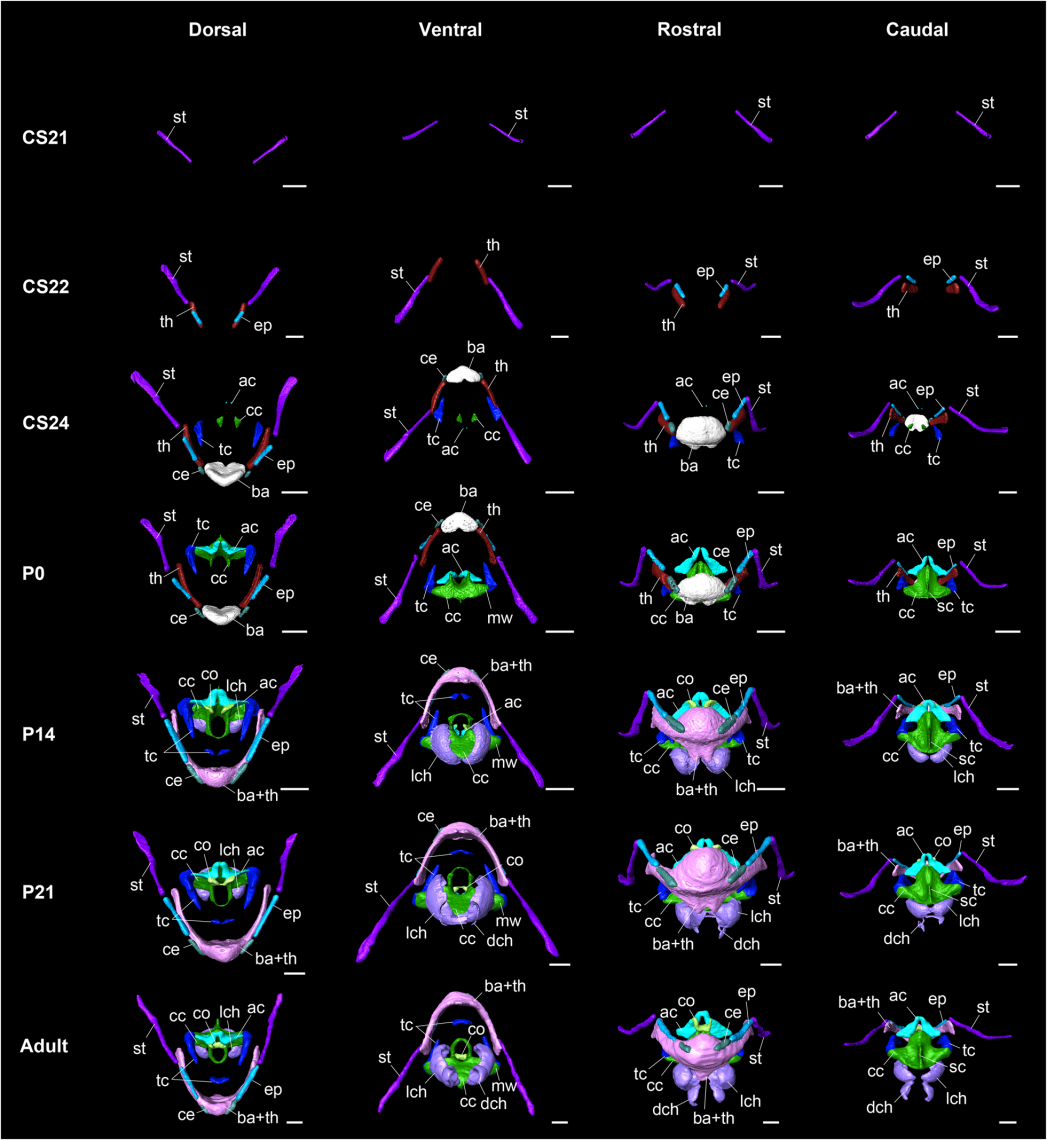
Three-dimensional reconstruction of the pre- and postnatal ossification and mineralization of the hyolaryngeal apparatus in horseshoe bats. Scale bars = 1 mm. See text for abbreviations. CS21: fetal specimen of *Rhinolophus pusillus*. The stylohyal is ossified. CS22: fetal specimen of *R. pusillus*. The thyrohyal and epihyal are ossified. CS24: fetal specimen of *R. pusillus*. The basihyal and thyrohyal are ossified. The arytenoid, cricoid, and thyroid cartilages are mineralized. P0: postnatal day 0 specimen of *R. pusillus*. The sagittal crest of the cricoid cartilage is mineralized. P14: postnatal day 14 specimen of *R. pusillus*. The lateral tracheal chambers are mineralized. P21: postnatal day 21 specimen of *R. pusillus*. The dorsal tracheal chambers are mineralized. Adult: adult specimen of *R. pusillus*

The presence of ossification of all craniocervical bony elements at each stage is summarized in Additional file 1: Tables S1–3. In horseshoe bats, ossification of the stylohyal occurred at mid-fetal stage (CS19) and other hyoid components did not start until late-fetal stage (CS21) (Fig. 7). The epihyal and thyrohyal were simultaneously ossified in the rostral region of the stylohyal in the CS22. In E18.5mice, the basihyal and ceratohyal regions ossified between the left and right thyrohyal and rostral regions of the epihyal region, respectively. Simultaneously, mineralization of the laryngeal cartilaginous elements, cricoid, thyroid, and arytenoid, became evident. The center of mineralization of the cricoid cartilage was observed in the proximal part of the cranial arch. The mineralization of the thyroid cartilage starts from the lateral part, at which point the future joint with the cricoid cartilage is formed. Mineralization of the arytenoid cartilage begins at the caudal tip of the corniculate process. In postnatal day 0 (P0) specimens of horseshoe bats, the mineralized cricoid cartilage further expanded to form muscular wings and a cricoid lamina with the sagittal crest. The proximal part of the cranial arch extended rostrally. The muscular wings of the cricoid cartilage were in contact with those of the thyroid cartilage. The small ossification center of the arytenoid cartilage in CS24 expanded to form corniculate and muscular processes immediately after birth. At P14, the stylohyal, epihyal, ceratohyal, and thyrohyal regions extend caudally. The thyrohyal and basihyal were fully fused and the suture was absent. The sagittal crest of the cricoid cartilage extended caudally. The cranial arch of the cricoid cartilage was further mineralized to fully cover the glottis. A pair of cranial cornua of the thyroid cartilage was mineralized caudally to the basihyal. The vocal process projected slightly at the ventral base of the arytenoid cartilage. The left and right dorsal tips of the corniculate processes were in contact with each other. From this stage onward, mineralization of the lateral tracheal chamber began. The lateral tracheal chamber was in contact with the ventral side of the cricoid cartilage, forming an internal cavity connected to the respiratory tract. The sagittal crest of the cricoid cartilage extended dorsally at P21. The left and right mineralized cranial cornua of the thyroid cartilage were merged. The left and right vocal processes of the arytenoid cartilage were also in contact with the median line. From P21 onward, the dorsal tracheal chamber began to mineralize. The mineralized portion of the dorsal tracheal chamber was in contact with the ventral portion of the lateral tracheal chamber. In adult horseshoe bats, the sagittal crest of the cricoid cartilage was sharper and thicker than that in juveniles. The mineralized lateral and dorsal tracheal chambers also thickened until sexual maturation.

**Fig. 8:**
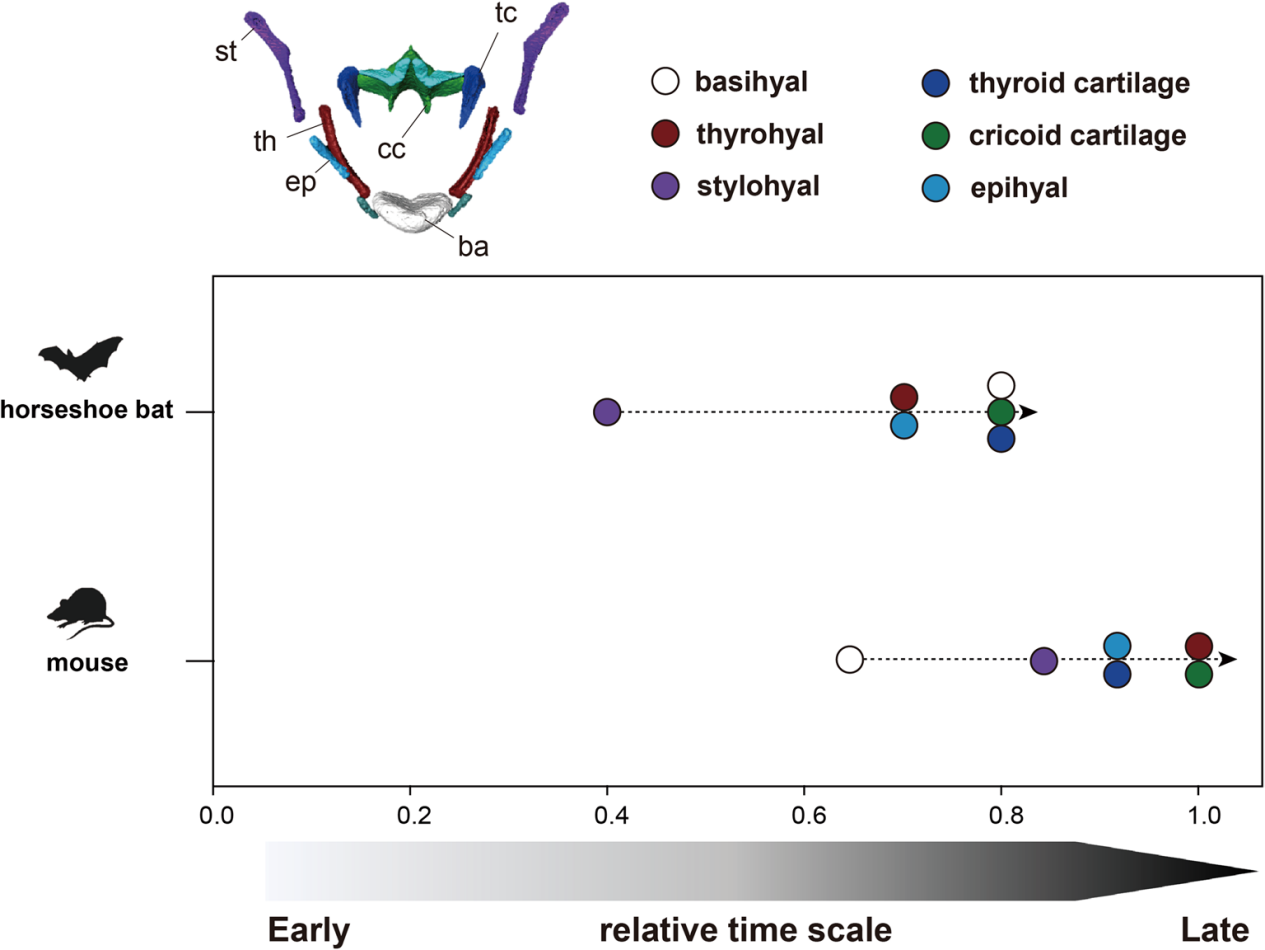
The relative ossification and mineralization timing of the bony and cartilaginous components of the hyolaryngeal apparatus in horseshoe bats (red) and laboratory mice (white). See text for abbreviations

**Fig. 9:**
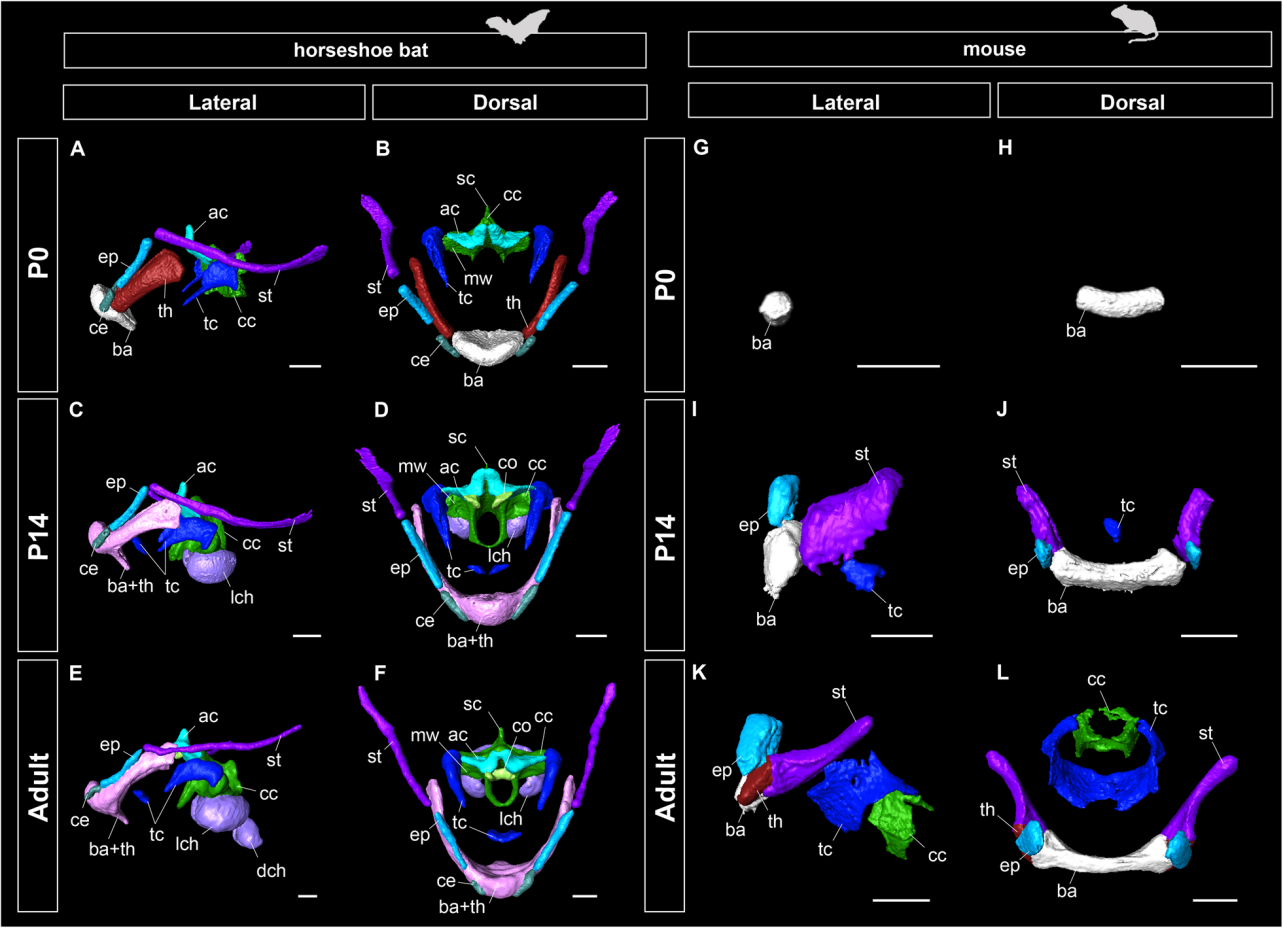
Three-dimensional reconstruction of postnatal ossification and mineralization of the hyolaryngeal apparatus in horseshoe bats (*Rhinolophus pusillus*) and laboratory mice (*Mus musculus*) based on micro-CT scanning. Scale bars = 1 mm. See text for abbreviations. **A** Lateral view of the hyolaryngeal apparatus of the postnatal day 0 specimen of *R. pusillus*. **B** Dorsal view of the hyolaryngeal apparatus of the postnatal day 0 specimen of *R. pusillus*. **C** Lateral view of the hyolaryngeal apparatus of the postnatal day 14 specimen of *R. pusillus*. **D** Dorsal view of the hyolaryngeal apparatus of the postnatal day 14 specimen of *R. pusillus*. **E** Lateral view of the hyolaryngeal apparatus of the adult specimen of *R. pusillus*. **F** Dorsal view of the hyolaryngeal apparatus of the adult specimen of *R. pusillus*. **G** Lateral view of the hyolaryngeal apparatus of the postnatal day 0 specimen of *M. musculus*. **H** Dorsal view of the hyolaryngeal apparatus of the postnatal day 0 specimen of *M. musculus*. **I** Lateral view of the hyolaryngeal apparatus of the postnatal day 14 specimen of *M. musculus*. **J** Dorsal view of the hyolaryngeal apparatus of the postnatal day 14 specimen of *M. musculus*. **K** Lateral view of the hyolaryngeal apparatus of the adult specimen of *M. musculus*. **L** Dorsal view of the hyolaryngeal apparatus of the adult specimen of *M. musculus*

Figure 7 shows the comparative anatomy of postnatal hyoid ossification and laryngeal mineralization in bats and mice. The relative ossification timing of the hyoid components is shown in Fig. 8 and Additional file 1: Table S3. While only the basihyal was ossified in P0 mice, all components of the hyoid apparatus started their ossification just after birth in bats. Indeed, except for the basihyal, almost all components of the hyoid apparatus ossified at an earlier stage in bats than in mice. Laryngeal mineralization was limited to the cricoid and thyroid cartilages in mice, whereas in bats, three laryngeal cartilages—the cricoid, thyroid, and arytenoid cartilages—were mineralized. Mineralization of the thyroid cartilage occurred in almost all parts of the mice, whereas in bats, the mineralized area of the thyroid cartilage was partial (Fig. 9A–F). Regarding the mineralization rate, laryngeal mineralization occurred in a relatively short period (P14-adult), while that of bats proceeded over the long term (CS24-adult).

### Allometric growth of the larynx and echolocation pulse ontogeny

**Fig. 10:**
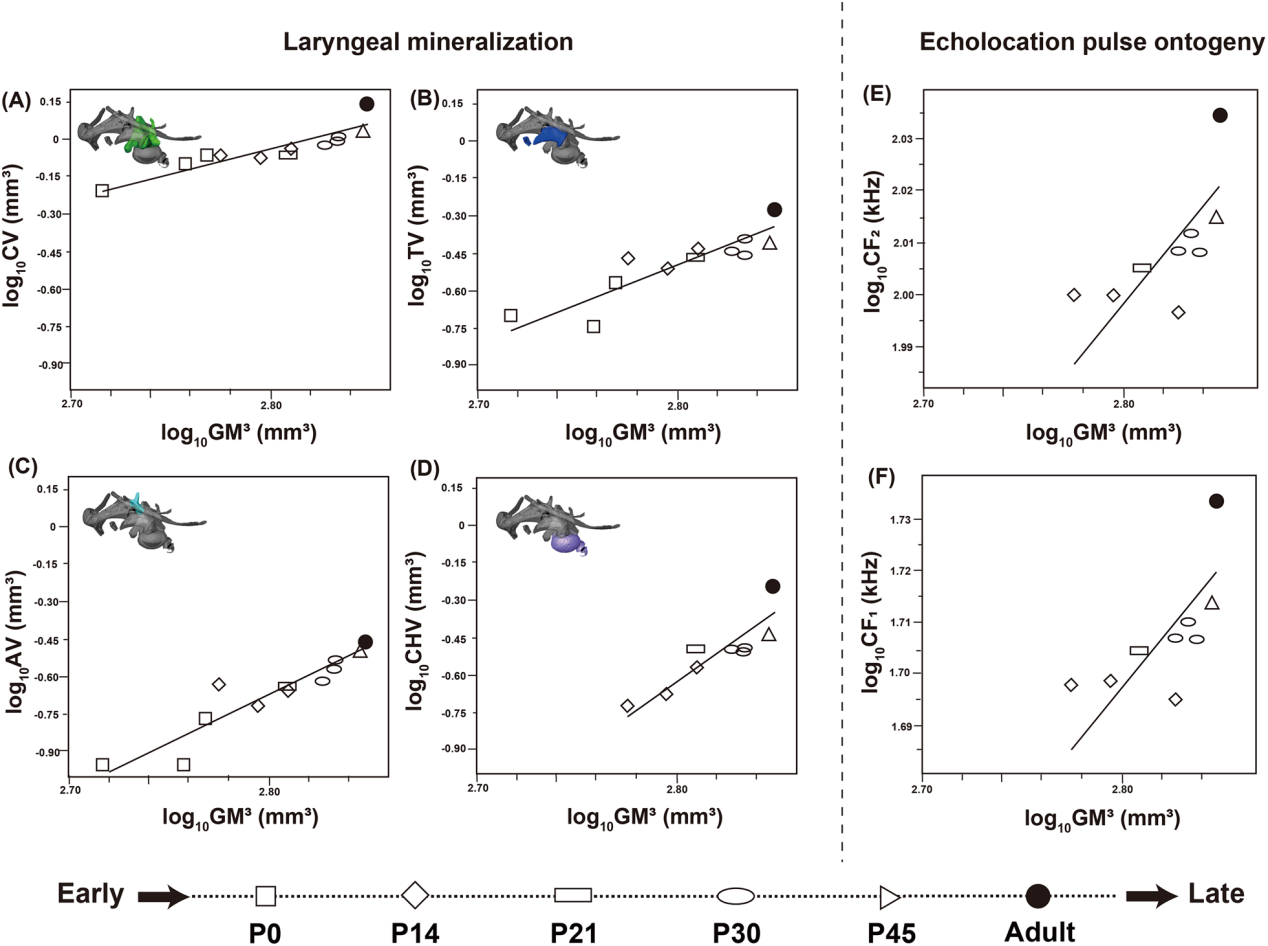
The regression analyses based on the reduced major axis between the mineralized volume of the laryngeal cartilages or pulse peak frequency against skull size (cubed geometric mean). **A** log_10_-transformed volume of the mineralized cricoid cartilage and log_10_-transformed GM^3^; **B** log_10_-transformed volume of the mineralized thyroid cartilage and log_10_-transformed GM^3^; **C** log_10_-transformed volume of the mineralized arytenoid cartilage and log_10_-transformed GM^3^; **D** log_10_-transformed volume of the mineralized tracheal chamber and log_10_-transformed GM^3^; **E** log_10_-transformed CF_2_ peak frequency and log_10_-transformed GM; **F** log_10_-transformed CF_1_ peak frequency and log_10_-transformed GM^3^. CV: cricoid cartilage volume; TV: thyroid cartilage volume; AV: arytenoid cartilage volume; CHV: tracheal chambers volume; GM^3^: cubed geometric mean of the skull height (SH), skull length (SL), and skull width (SW). Note that plots for P0 individuals are not shown in **D**, **E**, and **F**, as mineralization and echolocation pulse are absent at this stage

The basic statistics and results of the reduced major axis (RMA) regression analyses of mineralized laryngeal cartilage against the cubed geometric mean (GM^3^) of skull size in horseshoe bats are shown in Table 1 and Fig. 10A–D. A significant correlation with log_10_-transformed GM^3^ was detected in all log10-transformed volumes of mineralized laryngeal cartilage: cricoid cartilage (*r* = 0.90, *p* < 0.001), thyroid cartilage (*r* = 0.88, *p* < 0.001), arytenoid cartilage (*r* = 0.93, *p* < 0.001), and lateral tracheal chamber (*r* = 0.88, *p* < 0.001). The allometric exponents of all laryngeal cartilages were above 1.0 (i.e. positive allometry) as follows: 2.01 in the cricoid cartilage (95% CI from 1.21 to 2.83), 3.20 in the thyroid cartilage (95% CI from 1.82 to 4.39), 3.93 in the arytenoid cartilage (95% CI from 2.72 to 4.88), and 5.66 in the lateral tracheal chamber (95% CI from 3.14 to 7.69). The y-intercept values were − 5.68 in the cricoid (95% CI from − 7.95 to − 3.43), − 9.46 in the thyroid (95% CI from − 12.82 to − 5.59), − 11.68 in the arytenoid (95% CI from − 14.36 to − 8.23), and − 16.47 in the lateral tracheal chamber (95% CI from − 22.21 to − 9.36).

**Table 1:** Basic statistics of the allometric analyses of log10-transformed volume of the mineralized laryngeal cartilages or echolocation pulse, against log10-transformed geometric mean (GM)

	Slope (95%CI)	Intercept (95% CI)	*r*	*p*-value
Cricoid cartilage	2.01 (1.21 ~ 2.83)	− 5.68 (− 7.95 ~ − 3.43)	0.90	*p* < 0.0001
Thyroid cartilage	3.20 (1.82 ~ 4.39)	− 9.46 (− 12.82 ~ − 5.59)	0.88	*p* < 0.001
Arytenoid cartilage	3.93 (2.71 ~ 4.88)	− 11.68 (− 14.36 ~ − 8.23)	0.93	*p* < 0.001
Tracheal chamber	5.66 (3.14 ~ 7.69)	− 16.47 (− 22.21 ~ − 9.36)	0.88	*p* < 0.01
CF1	0.47 (0.04 ~ 0.73)	0.39 (− 0.36 ~ 1.61)	0.76	0.0165
CF2	0.46 (0.05 ~ 0.73)	0.70 (− 0.04 ~ 1.88)	0.76	0.0166

The results of the RMA regression analyses of the log10-transformed maximum frequency of the first-harmonic (CF_1_) and second-harmonic (CF_2_) pulses against the log10-transformed GM^3^ are shown in Fig. 10E–F. Significant correlations with the GM were detected for CF_1_ (*r* = 0.76, *p* < 0.05) and CF_2_ (*r* = 0.76, *p* < 0.05). The regression slope of both CF_1_ and CF_2_ significantly shows negative allometry as follows: 0.47 in CF_1_ (95% CI from 0.04 to 0.73) and 0.46 in CF_2_ (95% CI from 0.05 to 0.73). The y-intercept value was 0.39 in the CF_1_ (95% CI from − 0.36 to 1.61) and 0.70 in the CF_2_ (95% CI from − 0.04 to 1.88).

Figure 10 shows the ontogeny of the sonograms of the CF_1_ and CF_2_ pulses. The echolocation pulse was not confirmed until P13; thus, the biosonar pulse emission was acquired for at least 2 weeks after birth. At P14, the maximum frequencies of CF_1_ and CF_2_ were 49.58–49.99 kHz and 99.20–99.98 kHz, respectively. The values of CF_1_ and CF_2_ in P21 were 50.67 kHz and 101.23 kHz, respectively. In P30, the obtained values of each harmonic pulse were 50.89–51.28 kHz and 101.83–102.67 kHz. The maximum value of each harmonic pulse rose to 51.69 kHz and 103.37 kHz in P45. In adult individuals, the maximum frequencies of CF_1_ and CF_2_ finally reached 54.14 kHz and 108.25 kHz.

### Relationship between laryngeal mineralization and pulse ontogeny

**Fig. 11:**
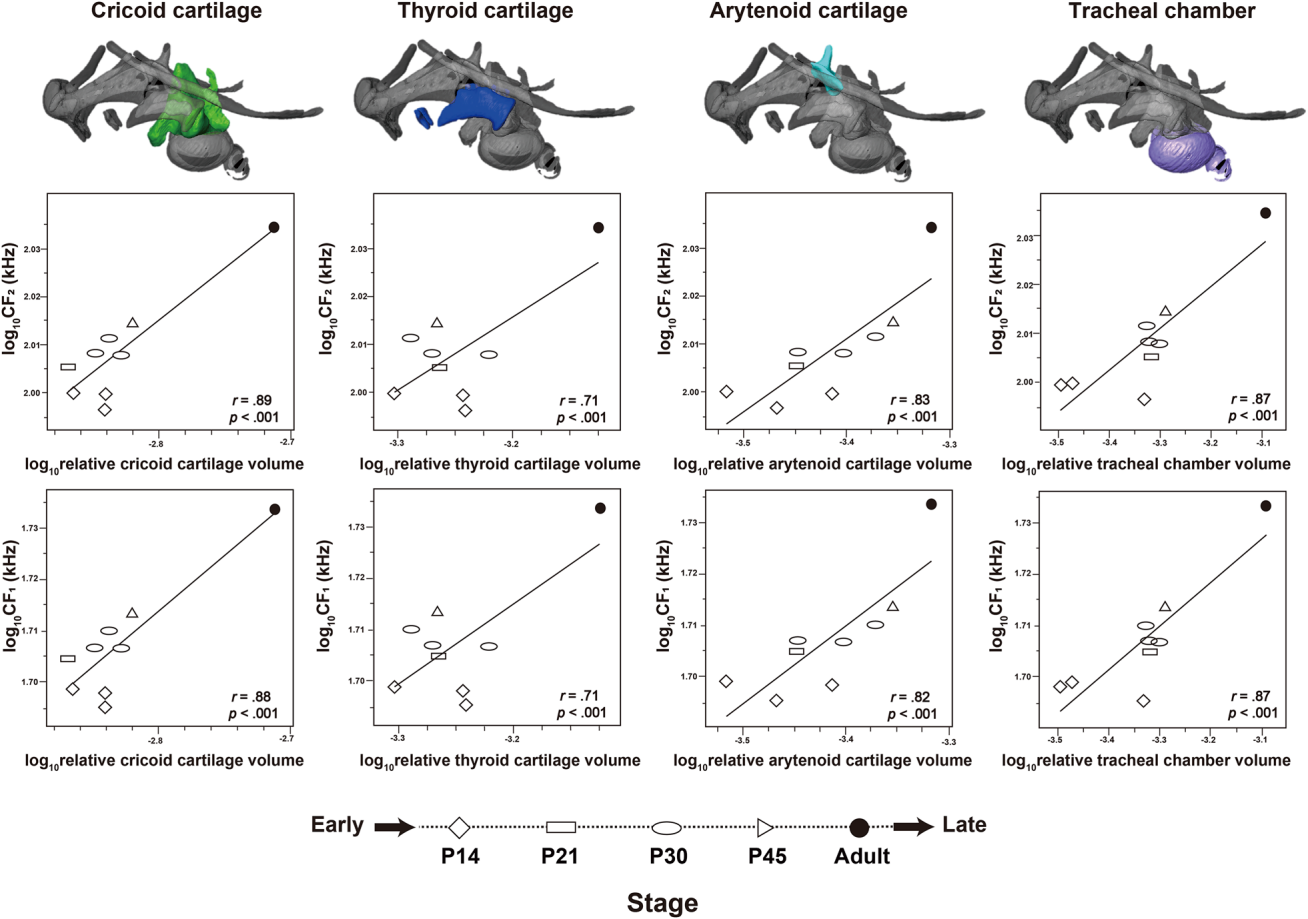
GLM analyses between the pulse peak frequency and relative volume of the mineralized laryngeal cartilages. Each volume was normalized by the cubed geometric mean of the skull height (SH), skull length (SL), and skull width (SW)

Table 2 and Fig. 11 illustrate the results of generalized linear model (GLM) analyses in horseshoe bats to investigate the relationship between the log10-transformed volume of the mineralized laryngeal cartilage and the log10-transformed peak frequencies of the CF_1_ and CF_2_ components of the echolocation pulse. Significant positive associations with the maximum frequency of the CF_1_ component were observed in the cricoid (slope = 0.21, standard error [SE] = 0.04, *p* < 0.001), thyroid (slope = 0.15, SE = 0.06, *p* < 0.001), arytenoid (slope = 0.15, SE = 0.04, *p* < 0.001), and lateral tracheal chambers (slope = 0.09, SE = 0.02, *p* < 0.001). Regarding the associations with the maximum frequency of the CF_2_ component, significant positive associations were detected in the cricoid cartilage (slope = 0.21, SE = 0.04, *p* < 0.001), thyroid cartilage (slope = 0.15, SE = 0.06, *p* < 0.001), arytenoid cartilage (slope = 0.15, SE = 0.04, *p* < 0.001), and lateral tracheal chamber (slope = 0.08, SE = 0.02, *p* < 0.001).

**Table 2:** Basic statistics of the generalized linear model analyses between log10-transformed volume of the mineralized laryngeal cartilages and log10-transformed peak frequency of CF_1_ or CF_2_

Laryngeal cartilage	Slope	Standard error of slope	Intercept	Standard error of intercept	*p*-value
CF1					
Cricoid cartilage	0.21	0.04	2.31	0.12	*p* < 0.0001
Thyroid cartilage	0.15	0.06	2.21	0.19	*p* < 0.01
Arytenoid cartilage	0.15	0.04	2.22	0.14	*p* < 0.001
Lateral tracheal chamber	0.09	0.02	1.99	0.06	*p* < 0.0001
CF2					
Cricoid cartilage	0.21	0.04	2.61	0.12	*p* < 0.0001
Thyroid cartilage	0.15	0.06	2.51	0.18	*p* < 0.01
Arytenoid cartilage	0.15	0.04	2.53	0.13	*p* < 0.0001
Lateral tracheal chamber	0.08	0.02	2.29	0.06	*p* < 0.0001

## Discussion

### General morphology of the hyolaryngeal complex in the horseshoe bats

The hyolaryngeal morphology of horseshoe bats has the following six unique characteristics compared to that of mice: (1) a spatial gap between the hyoid complex and thyroid cartilage; (2) enlarged intrinsic muscles (cricothyroid, thyroarytenoid, dorsal, and lateral cricoarytenoid muscles); (3) sagittal crest of the cricoid cartilage; (4) a fully separated dorsal cricoarytenoid muscle; (5) the presence of lateral and dorsal tracheal chambers, and (6) the additional branch of the cranial laryngeal nerve innervating the cricothyroid muscle. Characteristics (2), (3), and (5) are consistent with the descriptions of *Rhinolophus hipposideros* [44] and *Hipposideros caffer* [26]. As reported in other laryngeally echolocating bats [19, 50], the intrinsic laryngeal muscles of horseshoe bats are hypertrophied compared to those of mice, and the laryngeal cartilage is drastically modified to provide attachment sites for these muscles. The overall pattern of innervation of the intrinsic laryngeal muscles of horseshoe bats was identical to that of mice, except for a novel branch of the cranial laryngeal nerve and the absence of the foramen thyroideum (Fig. 3).

Our observations identified a hyoid–larynx gap, showing that the connection between the hyoid and larynx is evident in horseshoe bats (Fig. 1D). This structure has also been confirmed in other laryngeally echolocating bats such as *Desmodus rotundus* [34] and *Artibeus jamaicensis* [48]. Instead, the stylohyal bone is articulated with the ectotympanic bone, which contributes to the direct conduction of ongoing echolocation signals in laryngeally echolocating bats [34] and tree mice [16]. However, these gaps and articulations are absent in pteropodids (Giannini et al. [60]) and non-bat mammals (Fig. 1G). Therefore, in laryngeally echolocating bats, the hyoid apparatus itself is likely to be extended rostrocaudally, providing a gap between the hyoid apparatus and laryngeal cartilage and articulation between the stylohyal and ectotympanic. Biosonar sound requires rotation of the thyroid cartilage to increase the tension of the vocal folds with the superfast muscle [19]. Acoustic response analyses have revealed that the bony connection between the stylohyal and ectotympanic transfers sound signals at the most susceptible amplitude to the auditory bullae [40, 49]. Thus, as a result of the release from the tight connection, the hyoid complex and laryngeal cartilage might become mutually movable, providing higher flexibility for bone conduction and thyroid rotation, respectively.

The dorsal cricoarytenoid muscle was completely divided by the projection of the sagittal crest of the cricoid cartilage (Fig. 1C). In mice, the dorsal cricoarytenoid muscle was attached to the cricoid and arytenoid cartilages as a single muscle (Fig. 1G), whereas in bats, the separated left and right muscles were attached to the left and right arytenoid cartilages (Fig. 1C). This indicates that the separated dorsal cricoarytenoid muscle contracts independently, contributing to frequency adjustment in horseshoe bats. Generally, the dorsal cricoarytenoid muscle adducts with the arytenoid cartilage and adjusts the tension of the vocal folds during vocalization in mammals [57]. High-duty cycle (HDC) echolocating bats such as horseshoe bats emit sound signals multiple times per echolocation [22]. Previous studies have reported that the sagittal crest of the cricoid cartilage is also protruding in laryngeally echolocating lineages that emit calls orally (Mormoopidae [58] and Vespertilionidae [46, 59]), whereas that of the non-laryngeally echolocating bats, the pteropodids, is relatively smooth [60]. In contrast to rhinolophids (this study) and hipposiderids [26], the sagittal crests of the cricoid cartilages in these groups do not fully divide the dorsal cricoarytenoid muscles, indicating that this muscle pattern is unique to HDC-echolocating rhinolophoids. Therefore, the independent contraction of the dorsal cricoarytenoid muscle caused by the development of the sagittal crest of the cricoid cartilage would increase the abduction ficiency of the arytenoid cartilage during HDC pulse emission in horseshoe bats.

We confirmed that the cricothyroid muscle, possibly innervated by a novel branch of the cranial laryngeal nerve, had expanded toward the lateral tracheal chamber (Fig. 3). The lateral and dorsal tracheal chambers have been hypothesized to be key features involved in pulse emission through the nostrils in the genera *Rhinolophus* and *Hipposideros* in rhinolophoids and nycterids [26, 44]. Tracheal chambers have been theorized to function as Helmholtz resonators that increase sound intensity for laryngeal echolocation [26, 29]. Because biosonar pulse generation is underpinned by the superfast contraction of the cricothyroid muscle in laryngeally echolocating bats (Elemans et al. [19]), the expansion and contraction of the tracheal chambers are likely synchronized with that of the cricothyroid muscle. If the hypertrophied cricothyroid muscle and additional branches of the cranial laryngeal nerve are required for acoustic function in the tracheal chambers, this innervation pattern would be a synapomorphy of rhinolophid and hipposiderid bats. Further investigations on the innervation patterns of nycterids, rhinopomatids, and emballonurids that also have tracheal chambers (Denny [26]) are needed to test this hypothesis.

### Prenatal development of the hyolaryngeal complex in horseshoe bats and in mice

We found that the hyolaryngeal complex reached adult morphology in bats and mice at the early fetal stage (Figs. 3A, 4A). The bilaterally extended lesser and greater horns of the hyoid in horseshoe bats became evident at an earlier stage than those in CS17. In humans, the second pharyngeal cartilage forms the elongated lesser horn of the hyoid at Carnegie Stage 18, followed by the development of the greater horn of the hyoid [61]. These cases suggest that the elongation of each horn of the hyoid is due to chondrocyte proliferation during the early fetal stage. We also confirmed that the thyrohyoid muscle increasingly develops toward the rostral side and merges at the median line in mice but not in horseshoe bats (Fig. 4E, F). Around Carnegie Stage 20 in humans, the thyrohyoid muscle connects the greater horn of the hyoid and the thyroid cartilage [62], similar to rhinolophids. Together, the loosened hyoid–larynx connection of rhinolophids may result from differences in the initial construction of the thyrohyoid muscle, at least until CS17.

Our observations confirmed that each intrinsic laryngeal muscle became distinguishable at CS17 in bats and E14.5, in mice (Figs. 3 and 4). The time at which patterning of the intrinsic laryngeal muscles occurs in mice is consistent with the observations in the mouse embryo [63]. We also found that the left and right components of the dorsal cricoarytenoid muscle were present at the boundary of the sagittal crest, similar to CS17 (Fig. 4A). In mice, the dorsal cricoarytenoid muscle develops as a single muscle and is not divided by the sagittal crest throughout ontogeny (Fig. 4E–L). Because this pattern has also been described in rat embryos [64], a single dorsal cricoarytenoid muscle is likely to be conserved in rodents. In humans, the posterior cricoarytenoid muscle develops from two independent muscles and maintains a separate morphology until adulthood [65]. These facts suggest that the dorsal cricoarytenoid muscle of the horseshoe bat is not secondarily separated by the projection of the sagittal crest, but originally has two developmental centers. Despite less-documented myogenesis in non-model organisms, separated dorsal cricoarytenoid muscles in adults have been reported in elk, red deer [5], minipig [66], and horses [67]. Therefore, it is possible that a single dorsal cricoarytenoid muscle in mice is the derived phenotype in mammals, and the separated muscle in horseshoe bats is a synapomorphic trait in laurasiatherians.

We observed that the rostral tip of the cranial arch of the cricoid cartilage projected to split the cricothyroid muscle in horseshoe bats (Fig. 5D, G). The ventral component of the cricothyroid muscle was attached to the ventral side of the cranial arch, and the muscular wings of the cricoid cartilage were formed at CS20.5. Contrary to its development in mice, the ventral part of the cricothyroid muscle appeared to be newly formed and merged with the dorsal part (Fig. 5D). Therefore, the hypertrophied cricothyroid muscle in horseshoe bats may not be established by the expansion of a single component, but by the fusion of the original cricothyroid muscle and novel muscle components. Considering that this muscle is also attached to the lateral tracheal chamber, this pattern may be unique to rhinolophid and hipposiderid bats. The cricothyroid muscle of laryngeally echolocating bats has been reported to be hypertrophied [26] and capable of high-speed contraction in a few species (*Myotis daubentonii* [19] and *Myotis lucifugus* [68]). Although the early development of the cricothyroid muscle has not yet been documented in other laryngeally echolocating lineages, the present study implies that the hypertrophied cricothyroid muscle is established through different developmental pathways among lineages with different biosonar pulse generation capabilities. Moreover, combined with observations in postnatal individuals (Fig. 1), the development of the cricothyroid muscle may proceed until birth. A similar pattern has been reported in horseshoe petrosal, in which the size growth is unusually prolonged until sexual maturation, leading to a remarkably enlarged cochlea in horseshoe bats [37]. The present study implies that the development of multiple organs related to both the reception and emission of biosonar sounds is prolonged concomitantly with the sophistication of laryngeal echolocation in horseshoe bats.

### On the embryonic origins of the tracheal chambers in horseshoe bats

We have described the early development of the lateral and dorsal tracheal chambers in horseshoe bats (Fig. 6). Immunohistochemistry for the Sox9 protein identified that the lateral and dorsal tracheal chambers developed by chondrocyte proliferation, such as the laryngeal cartilage (Fig. 6A-2). It has been hypothesized that tracheal chambers are formed by the modification of the tracheal ring (Denny et al. [26]). Histological observations indicated that the condensed chondrocyte mass comprising the lateral tracheal chamber originated from the ventral portion of the cricoid cartilage, whereas the tracheal rings had not yet chondrified (Fig. 6A). Thus, we suggest that the lateral and dorsal tracheal chambers are cricoid-derived laryngeal cartilages acquired in rhinolophids and hipposiderids due to the expansion of the caudal tip of the cricoid cartilage. It has been widely accepted that the rhinolophids and hipposiderids significantly radiate their bioacoustics strategy, as represented by the emission of HDC pulses [22] and DSC, allowing them to improve the accuracy of target localization [31]. Tracheal chambers are known to function as pulse amplifiers for the reinforcement of sound intensity because of their susceptibility to attenuation during emission through the nostrils [26, 29]. The concept of “key innovations” has been defined in evolutionary developmental biology as a substantial change of the characters that have triggered lineage diversification [69, 70]. In accordance with this definition, tracheal chambers found explicitly in laryngeally echolocating bats emitting pulses with their nostrils (rhinolophids, hipposiderids, nycterids, rhinopomatids, and emballonurids) should be regarded as key innovations in the larynx. In light of this, key innovations in the cricoid cartilage might have led to the sophistication of laryngeal echolocation in tandem with the acquisition of HDC and DSC capabilities.

Genetic studies have reported that the development of respiratory organs such as the larynx, trachea, and lungs is regulated by the expression of *Fgf10* [71], *chordin* [72], *Fuz*, *Gli3* [73], *β-catenin* [74], and *Tbx1* [75]. In particular, it has been reported that *Fgf10* knockout experiments induce the disorganization of mesenchymal cells at the distal end of the trachea and the absence of lung buds [71]. Given this, changes in the genetic factors represented by *Fgf10* might result in changes in the distribution of mesenchymal cells, thereby inducing the expansion of the cricoid cartilage in horseshoe bats. Further investigations using gene expression analysis are required to uncover the genetic basis underlying the evolution of tracheal chambers.

### Pre- and postnatal ossification of the hyolaryngeal apparatus

This study described hyoid ossification (Figs. 7 and 9) and estimated the relative ossification timing of each element (Fig. 8). The timing of ossification of the hyoid components in horseshoe bats was accelerated relative to that of mice, except for the basihyal component (Fig. 8), although hyoid condensation was simultaneously observed at the early fetal stage in both species (Fig. 4). This indicates that the ossification sequence was not necessarily consistent with the chondrification sequence. In mice, the basihyal was the only hyoid component to ossify at birth (Fig. 9G), whereas in horseshoe bats, all bony elements in the hyoid apparatus were already ossified (Fig. 9A). In laryngeally echolocating bats, the stylohyal bone is articulated with an ectotympanic [34], which possibly facilitates the transmission of the ongoing signal into the inner ear through bone conduction [42]. A recent biomechanical analysis revealed that the closer the bony element was to the basihyal bone, the higher the sound pressure is (Snipes and Carter [42]). The ongoing echolocation pulse, which is produced in the larynx, transfers to the thyrohyal and basihyal regions and subsequently propagates into the ceratohyal, epihyal, stylohyal, and ectotympanic regions. Our results suggest that construction of the stylohyal-ectotympanic unit is preceded relatively by the thyrohyal–basihyal unit.

Our analyses revealed that the thyroid, cricoid, and arytenoid cartilages began mineralization immediately before birth in horseshoe bats. The cricoid and arytenoid cartilages were entirely mineralized, whereas mineralization of the thyroid cartilage was limited to the lateral and rostral parts, even in the adult stage (Fig. 7). The pattern of laryngeal mineralization in horseshoe bats contrasts with that in phyllostomid bats, which possess non-mineralized thyroid and arytenoid cartilages [26, 40, 46]. It has been suggested that laryngeal cartilage, which requires dynamic motion, tends to be more mineralized than static cartilages such as the cricoid cartilage [46]. Horseshoe bats produce high-intensity sounds in contrast to phyllostomids that emit low-intensity pulses [76]; thus, the highly mineralized cricoid and arytenoid cartilages in horseshoe bats would provide physical support for the superfast contraction of the hypertrophied cricothyroid muscle. Considering that the mineralized lateral and rostral parts of the thyroid cartilage correspond to the attachment sites of the cricothyroid and thyroarytenoid muscles, respectively (Fig. 1A–D), limited mineralization may be due to the high functional requirements of these two muscles for laryngeal echolocation. The limited mineralization of the thyroid cartilage, on the other hand, may be due to the high functional requirements of the cricothyroid and thyroarytenoid muscles for pulse generation. Indeed, phyllostomid bats without mineralized thyroid and arytenoid cartilage possess a relatively smaller cricothyroid muscle than other echolocating bats [28, 45]. Taken together, the differences in the mineralized laryngeal cartilage between the rhinolophids and hipposiderids groups and phyllostomids may imply that these two nasally echolocating lineages employ different morphologies and kinetics of the laryngeal muscle during laryngeal echolocation.

Mineralization of the lateral and dorsal tracheal chambers began at P14 and P21, respectively (Figs. 6E, F, 7 B, C). Although the tracheal chambers do not have attachment sites for the other muscles, except for the cricothyroid muscle, almost all the tracheal chambers were mineralized, such as the cricoid and arytenoid cartilages. Chondrification was already evident at CS17 (Fig. 6A), and connections between the lateral tracheal chamber and cricoid cartilage and between the dorsal tracheal chamber and third tracheal ring were established until CS22 (Fig. 6B). The air sac in the tracheal chambers prevents the echolocation pulse from being reflected from the lungs toward the auditory organs [77]. The tracheal chambers would vibrate during the emission of the biosonar pulse and are exposed to mechanical stress, thereby leading to mineralization of the entire cartilage. Following the mineralization of the other laryngeal cartilages, the structurally strengthened cartilages would increasingly reinforce the capability of biosonar pulse emission.

### Relationship between development and pulse ontogeny

**Fig. 12:**
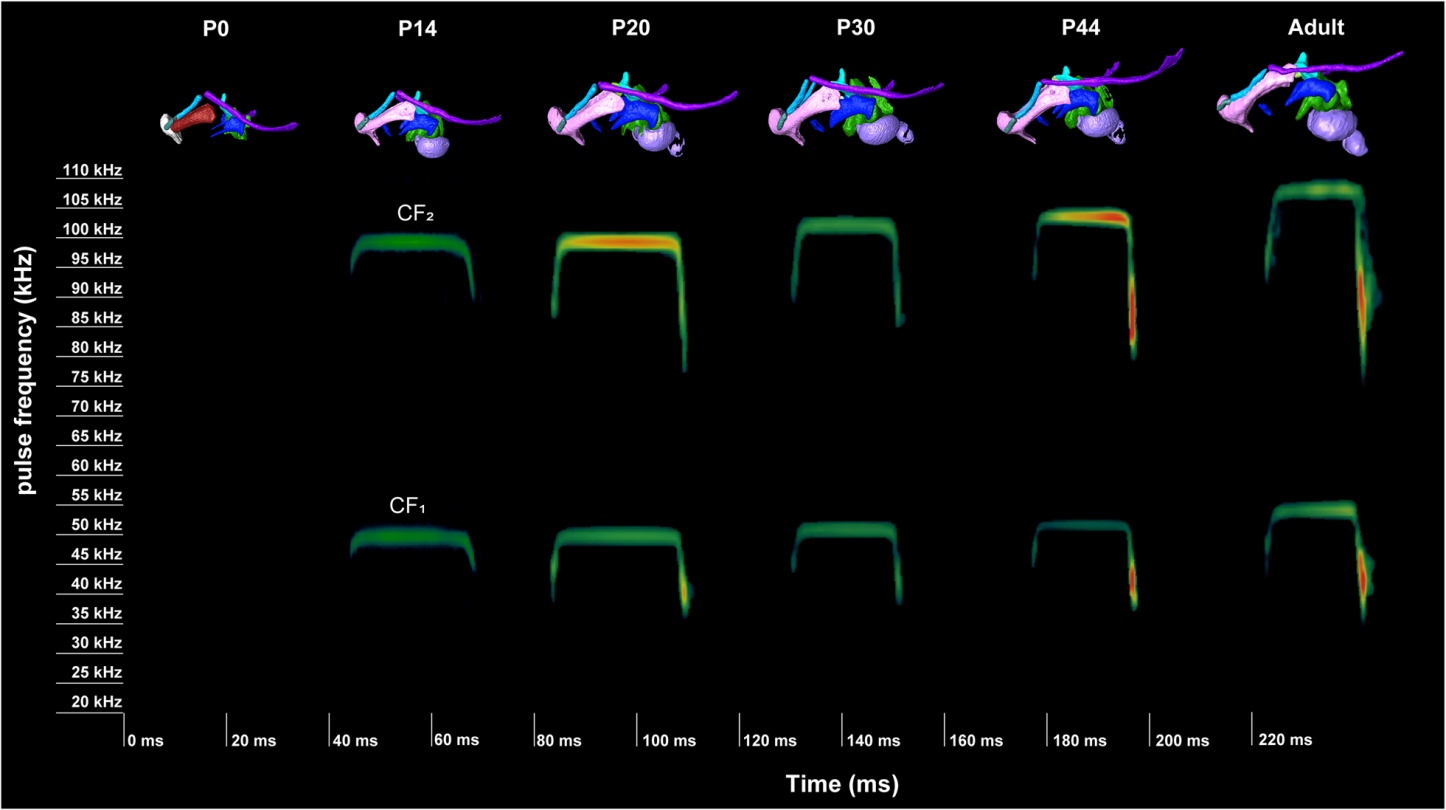
Ontogeny of the hyolaryngeal apparatus morphology and the echolocation pulse in *Rhinolophus pusillus*

We confirmed that no echolocation pulses were observed at the newborn (P0) stage (Fig. 12), as reported in a previous study [78]. In the P0 specimen, the basihyal bone was not fused with the thyrohyal bone, and each component of the lesser horn (ceratohyal, epihyal, and stylohyal) of the hyoid was not in contact, indicating that the capability of bone conduction was still immature at this stage (Figs. 6D, 7A). Furthermore, mineralization of the rostral part of the thyroid cartilage, cranial arch of the cricoid cartilage, and lateral tracheal chamber was not observed at this stage. Because the basihyal–thyrohyal unit is most susceptible to sound pressure during laryngeal echolocation [42], the establishment of a strongly supported hyoid apparatus may be essential for the acquisition of biosonar pulse emission. The echolocation pulse is known to gradually develop from spontaneous calls used for mother–infant communication when juvenile individuals start powered flights [79]. Our findings suggest that the progression of laryngeal mineralization reinforces the rapid contraction of the intrinsic laryngeal muscles and consequently allows bat pups to distinguish the echolocation pulse from precursor calls. As tracheal chambers improve the sound intensity for laryngeal echolocation [26, 29, 77], well-mineralized tracheal chambers are required for the maturation of high-intensity pulses in horseshoe bats [1]. Therefore, in contrast to phyllostomids with low-intensity calls, the structural demand for the endurance of high-intensity signals is likely reflected in the progressive mineralization of the laryngeal cartilages of horseshoe bats.

We detected positive allometric patterns in all laryngeal cartilages compared to the cubed geometric mean (Fig. 10A–D). Regarding the cricoid cartilage, mineralization occurred within a short period immediately before birth (Fig. 10A). The cricoid cartilage is surrounded by the dorsal cricoarytenoid and cricothyroid muscles (Fig. 1B–D). We suggest that the precedence of the mineralization of the cricoid cartilage allows horseshoe bats to promptly reinforce the foundation of their superfast muscles. Moreover, the regression slope of the peak frequency of the echolocation pulse against the cubed geometric mean showed a negative allometric pattern (Fig. 10E, F). The correlation between body mass and sound frequency in mammals has been widely recognized as size–frequency allometry [80]. A previous study reported a significant positive relationship between forearm length and the frequency of the second harmonic pulse in the juvenile stage of *R. cornutus* [79] and *R. ferrumequinum* [81], but the negative allometric pattern from juvenile to adult stages found in the present study (Fig. 10) indicates that the pulse peak frequency does not necessarily increase at a rate similar to that of body size.

Our GLM analyses identified significant correlations between the cricoid thyroid, arytenoid cartilage, and tracheal chambers and the peak frequencies of the CF_1_ and CF_2_ components (Fig. 11). Since the mineralized parts of the laryngeal cartilage correspond to the attachment sites of the cricothyroid, thyroarytenoid, and cricoarytenoid muscles (Fig. 1A–D), these four laryngeal cartilages are likely to be highly susceptible to mechanical stress during muscle contraction. The generation of high-frequency sounds requires superfast muscle (i.e., the cricothyroid muscle) control of the vocal cords [19]. Abduction of the glottis to vibrate the vocal fold involves contraction of the dorsal cricoarytenoid muscle. It has been suggested that tracheal chambers contribute to sound intensity [26, 29, 52, 77]. Given that the ventral part of the cricothyroid muscle is attached to the lateral tracheal chambers, mineralization of the tracheal chambers can be promoted by superfast contraction of this muscle. Thus, the ontogeny of the pulse peak frequency was also reflected in the mineralization of the tracheal chambers. Hence, we suggest that the ontogeny of the peak frequency of the echolocation pulse is underpinned by the mineralization of these four laryngeal cartilages in horseshoe bats.

### Implications for the evolution of the laryngeal echolocation in bats

We found that horseshoe bats possess a unique morphology, such as hypertrophied intrinsic laryngeal muscles (Fig. 1B) and a sagittal crest (Fig. 1C), as reported in previous studies [26, 44]. Regarding hyoid morphology, we identified that horseshoe bats possess the following five hyoid elements: basihyal, thyrohyal, ceratohyal, epihyal, and stylohyal (Figs. 1A–D, 3A–C, 6, 7). A novel pattern of innervation of the cranial laryngeal nerve was also identified using immunohistochemical staining (Fig. 3). Considering that the novel branch of the cranial laryngeal nerve runs into the ventrally expanded portion of the cricothyroid muscle, the hypertrophied cricothyroid muscle comprises two components: (1) homologous cricothyroid muscle in general mammals and (2) novel muscle acquired along with the acquisition of the tracheal chambers in horseshoe bats (Fig. 3). Our findings corroborate that the evolution of sophisticated biosonars, such as DSC, in horseshoe bats might be underpinned by morphological changes in the hyolaryngeal complex.

Hyoid morphology has been recognized as a key component of laryngeal echolocation [33, 34, 39, 42, 43, 47, 52, 58, 82–85]. Regarding the entire morphology of the hyoid apparatus in bats, it has been suggested that the number of hyoid components varies among lineages [45]. In contrast to the hyoid apparatus with the segmented lesser cornua in horseshoe bats (Figs. 1A–D, 6, 7A–C), for example, vespertilionid bats do not have the epihyal and ceratohyal, forming the lesser cornua only with the stylohyal bone [86]. The genus *Myotis*, on the other hand, is known to have a lesser cornua formed by the two bony elements [86], although the homology of each component in horseshoe bats is still unclear. Because morphological differences in the hyoid apparatus are related to variations in the efficiency of sound propagation [42], morphological changes in the hyoid apparatus may contribute to the diversification of acoustic properties, such as sound frequency, duration, and intensity, in laryngeally echolocating bats.

The present study identified unique characteristics such as a possibly novel branch of the cranial laryngeal nerve in rhinolophids. This lineage may have achieved pulse generation with different laryngeal kinetics of the superfast muscle compared to that of yangochiropterans [19]. Regarding pteropodids, the sister clade of the rhinolophoids within the yinpterochiropterans, the morphology of their laryngeal muscles has been reported to be similar to that of the vespertilionid bats in the yangochiropterans, rather than that of the rhinolophoids [52, 59]. Given this, laryngeal development may also be divergent within lineages with different echolocation capabilities, as well as the petrosal and stylohyal (Nojiri et al. [39]). The molecular study detected positive selection of the hearing genes of the ancestral branches of bats, suggesting that the common ancestor of bats employed primitive sonar, such as the lingual echolocation of the genus *Rousettus*, and then rhinolophoids and yangochiropterans acquired sophisticated laryngeal echolocation [87]. To date, there have been few studies on laryngeal development in bats, especially in pteropodids and yangochiropterans, making this hypothesis difficult to validate. This study reveals several key characteristics involved in the generation of echolocation pulses. We identified significant differences in the morphology of the mineralized laryngeal cartilage and the presence of the echolocation pulse between P0 and P14 specimens. For example, the emission of the echolocation pulse was confirmed after P14, at which the lateral tracheal chambers started mineralization (Fig. 9C, D), possibly contributing to the acquisition of the echolocation pulse production capability. Given this, the macroevolutionary changes in the cricoid cartilage and acquisition of tracheal chambers might have co-evolved to some extent with the acquisition of sophisticated biosonar in horseshoe bats. In subsequent studies, investigations of the commonality and disparity of hyolaryngotracheal morphology in extinct and extant bats would provide key insights into the evolutionary origins of laryngeal echolocation and diversification of biosonar in bats.

## Conclusions

Three-dimensional reconstruction of the hyolaryngeal apparatus revealed the unique morphology of the cricoid cartilage, intrinsic laryngeal muscle, and cranial laryngeal nerve in horseshoe bats. The present study suggests that the sagittal crest of the cricoid cartilage and the separated dorsal cricoarytenoid muscles may be key features involved in echolocation pulse generation. Laryngeally echolocating bats are characterized by hypertrophied intrinsic laryngeal muscles, including the cricothyroid muscles, with superfast contractions. We provide a new perspective that the hypertrophied cricothyroid muscle of horseshoe bats comprises homologous muscles in mammals and possibly novel components acquired in rhinolophids and hipposiderids, which may have served as key innovations in the adaptive radiation of these HDC bats. This drastic morphological innovation is possibly in concert with the acquisition of tracheal chambers, allowing them to conduct sophisticated biosonar with high accuracy for target localization. In addition, we found that the maximum frequency of the echolocation pulse was significantly correlated with mineralization of the cricoid and arytenoid cartilages. Given the acoustic diversity in laryngeally echolocating bats, it is possible that the postnatal mineralization of each laryngeal cartilage may independently regulate each acoustic property. Future studies integrating the hyolaryngeal development of pteropodids, yangochiropterans, and other rhinolophoids will offer insights into the relationship between variations in sound properties and hyolaryngeal morphology and improve our understanding of how morphological changes in the hyolaryngeal apparatus shape acoustic diversity in bats.

## Methods

### Data acquisition

A total of 32 specimens of the genus *Rhinolophus* species (*R. pusillus*, *n* = 28; *R. malayanus*, *n* = 4) and ten specimens of C57/BL6 mice (*Mus musculus*) were studied. The fetal and postnatal specimens of *R. pusillus* were collected by the authors in Japan and Vietnam. *Rhinolophus pusillus* from Japan was collected under the permission granted by the Gosen Local Government in Japan (No. 1–4-2021) and is stored at the University of Tsukuba (UT). The fetal specimens of *R. malayanus* were collected by the authors during field sampling in Vietnam under permission granted by the An Giang Province People’s Committee in Vietnam (No. 497/VPUBND-NC and No. 5893/VPUBND-KTN) and the Vietnam Administration of Forest, belonging to the Ministry of Agriculture and Rural Development (No. 1072/TCLN-BTTN and No.326/TCLN-BTTN). *Rhinolophus pusillus* from Japan and Vietnam are considered to compose a single species, and geographic variation of the hyolaryngeal morphology was not detected between individuals from Japan and those from Vietnam. Our postnatal samples of *R. pusillus* comprehensively covered the overall ontogeny of the echolocation pulse, but some stages of prenatal specimens were lacking to describe the ossification sequence of the hyolaryngeal bony components. Therefore, fetal specimens of *R. malayanus*, both of which are members of the same genus *Rhinolophus* [88], were additionally included. The examined specimens of *R. pusillus* and *R. malayanus* were first confirmed to show no significant difference in qualitative hyolaryngeal morphology. Animal ethics for mice sampling and experiments were approved at Tokyo Medical and Dental University, Japan (A2019-060C3 and A2021-198A). All experiments were conducted following the ARRIVE guidelines following the Japanese law on animal welfare. All pregnant individuals captured in this study were euthanized by isoflurane inhalation. Fetal bat specimens were staged following Cretekos Stage (CS) [89]. The postnatal stage of bats was estimated by the time when the first birth was observed in the reproductive colony. The details of all specimens are summarized in Table 3. Fetal specimens examined in this study include stages from CS16 to CS24 (*R. pusillus*: CS16, 17, 18, 19, 20, 21, 22, 23, 24; *R. malayanus*: CS18, 20.5, 22). Postnatal specimens, all of which were *R. pusillus*, covered postnatal day 0, 14, 21, 30, 45, and adult. One adult individual of *R. pusillus* was loaned from the curatorial collection held in Kanagawa Prefectural Museum of Natural History (KPMNH), and all samples of *R. malayanus* from the curatorial collection held in the Institute of Ecology and Biological Resources, Vietnamese Academy of Science and Technology (IEBR). All mouse fetuses from E14.5 to E18.5 and postnatal specimens from P0 to adult (*n* = 10) were collected at Tokyo Medical and Dental University (TMDU). Pregnant mice were killed by cervical dislocation. The collected specimens were fixed using a mixture of ethyl alcohol:acetic acid:formalin (6:3:1) and stored in 70% ethyl alcohol. Specimens used in this study are summarized in Table 3.

**Table 3:** All specimens examined in this study

Species	ID	Storage	Stage	Bone micro-CT	diceCT	Tissue section
*Mus musculus*	NT22-002	TMDU	E14.5			x
*Mus musculus*	NT22-003	TMDU	E16.5			x
*Mus musculus*	JP19-001	TMDU	E17.5	x		
*Mus musculus*	NT22-004	TMDU	E18.0			x
*Mus musculus*	NT22-005	TMDU	Postnatal day 0	x		
*Mus musculus*	NT22-011	TMDU	Postnatal day 0		x	
*Mus musculus*	NT22-007	TMDU	Postnatal day 7	x		
*Mus musculus*	NT22-008	TMDU	Postnatal day 14	x		
*Mus musculus*	NT22-012	TMDU	Postnatal day 14		x	
*Mus musculus*	NT22-010	TMDU	Adult	x		
*Rhinolophus pusillus*	JP20-052	UT	CS16	x		
*Rhinolophus pusillus*	JP20-044	UT	CS17	x		
*Rhinolophus pusillus*	JP20-055	UT	CS17			x
*Rhinolophus pusillus*	JP20-039	UT	CS18	x		
*Rhinolophus pusillus*	QN043	IEBR	CS19	x		
*Rhinolophus pusillus*	QN041	IEBR	CS20	x		
*Rhinolophus pusillus*	B160413-4	IEBR	CS21	x		
*Rhinolophus pusillus*	JP21-032	UT	CS22	x	x	
*Rhinolophus pusillus*	XL2016-25	IEBR	CS22	x		
*Rhinolophus pusillus*	VN17-299	IEBR	CS23	x		
*Rhinolophus pusillus*	B200413-7	IEBR	CS24	x		
*Rhinolophus pusillus*	JP21-047	UT	Postnatal day 0	x		
*Rhinolophus pusillus*	JP21-073	UT	Postnatal day 14	x		
*Rhinolophus pusillus*	JP21-074	UT	Postnatal day 14	x		
*Rhinolophus pusillus*	JP21-075	UT	Postnatal day 14	x	x	
*Rhinolophus pusillus*	JP22-067	UT	Postnatal day 14	x		
*Rhinolophus pusillus*	JP22-068	UT	Postnatal day 14	x		
*Rhinolophus pusillus*	JP22-082	UT	Postnatal day 21	x		
*Rhinolophus pusillus*	JP21-083	UT	Postnatal day 21	x		
*Rhinolophus pusillus*	JP21-091	UT	Postnatal day 21		x	
*Rhinolophus pusillus*	JP21-100	UT	Postnatal day 30	x	x	
*Rhinolophus pusillus*	JP21-101	UT	Postnatal day 30	x		
*Rhinolophus pusillus*	JP21-104	UT	Postnatal day 45	x	x	
*Rhinolophus pusillus*	JP21-025	UT	Adult		x	
*Rhinolophus pusillus*	KPMNH2937	IEBR	adult	x		
*Rhinolophus malayanus*	VN20-032	IEBR	CS18	x		
*Rhinolophus malayanus*	VN20-054	IEBR	CS18	x		
*Rhinolophus malayanus*	VN19-057	IEBR	CS20.5			x
*Rhinolophus malayanus*	VN19-029	IEBR	CS22			x

### Three-dimensional reconstruction of hyolaryngeal morphology

**Fig. 13:**
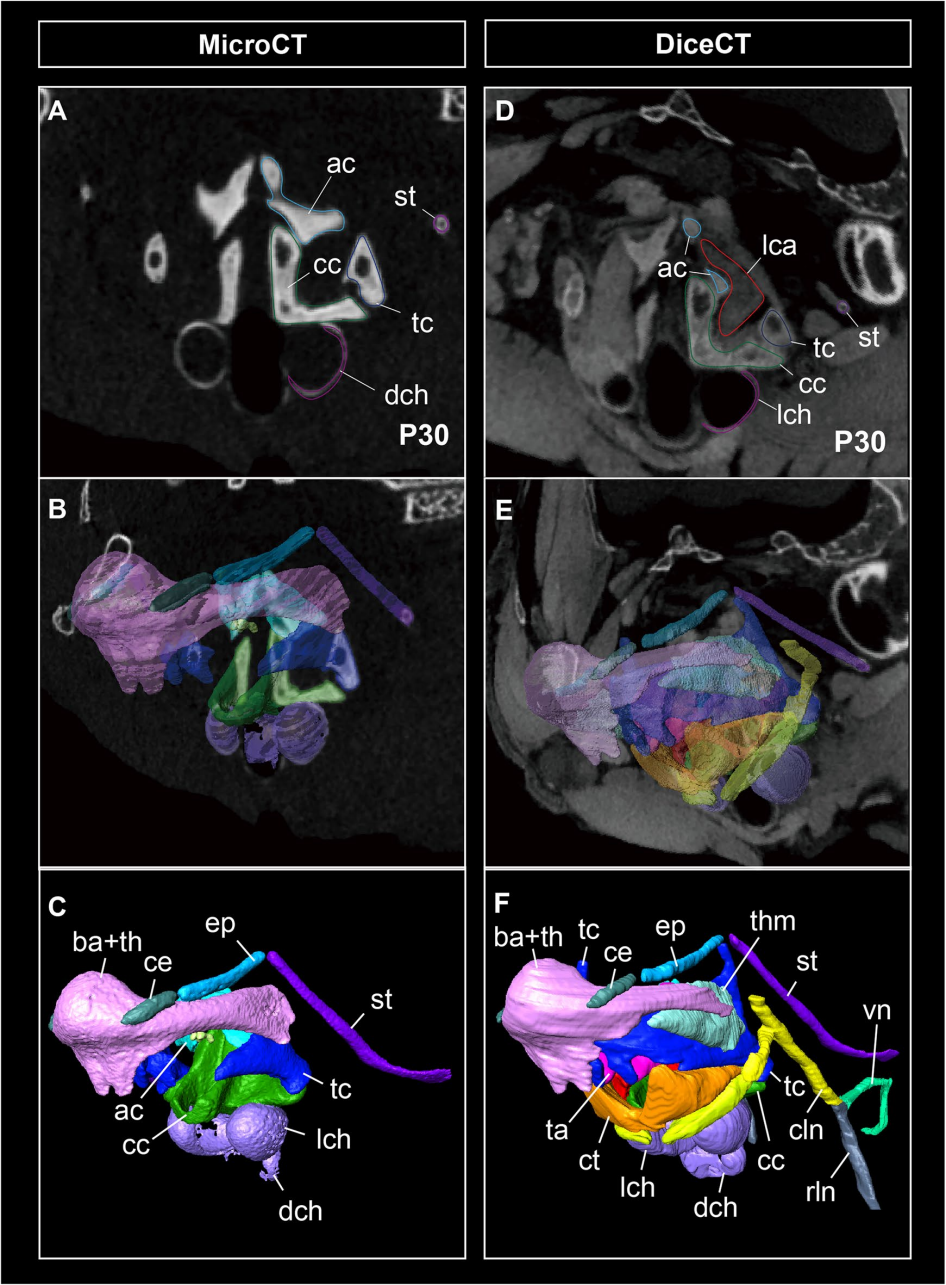
The three-dimensional reconstruction procedure of the hard tissue and soft tissue of the hyolaryngeal apparatus in postnatal day 30 of *Rhinolophus pusillus*. **A** One example of the tomographic image obtained from micro-CT scanning without staining. **B** Three-dimensionally reconstruction process of the morphology of the hyolaryngeal cartilages with the tomographic images. **C** Three-dimensionally reconstructed morphology of the hyolaryngeal cartilages. **D** One example of the tomographic image obtained from diffusible iodine-based contrast-enhanced CT method. **E** Three-dimensionally reconstruction process of the morphology of the hyolaryngeal cartilages with the tomographic images. **F** Three-dimensionally reconstructed morphology of the hyolaryngeal cartilages, intrinsic laryngeal muscles, and vagus nerve (cranial nerve X). Left side of the hyolaryngeal morphology is shown

**Fig. 14:**
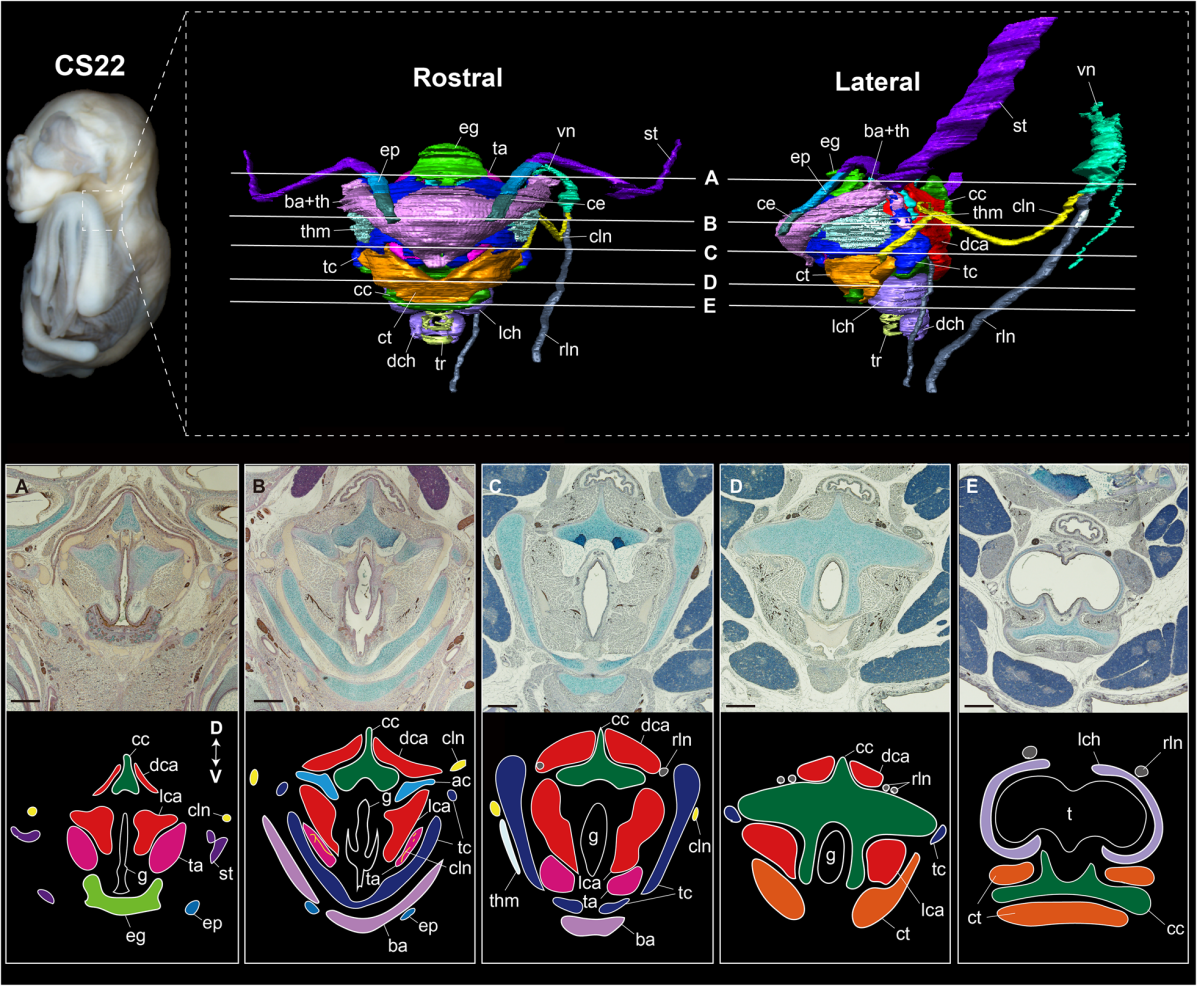
The three-dimensional reconstruction procedure of the gross anatomy of the hyolarynx of the bat fetus (CS22; *Rhinolophus pusillus*) with serial tissue sections. All sections were immunostained with the acetylated tubulin antibody to visualize the nerves. Left side of the hyolarynx is shown in the lateral view. After immunostaining, all sections were additionally stained with alcian blue and hematoxylin. Scale bars = 500 μm. See text for abbreviations

To investigate the development of the hyolaryngeal apparatus in the fetal and postnatal stages, we used microcomputed tomography (micro-CT) at University of Tsukuba (inspeXio SMX-90CT Plus, Shimadzu Corporation, Tokyo) and University Museum of University of Tokyo (TXS225-ACTIS, Tesco, Tokyo) with a 70 kV source voltage and 100 mA source current. To visualize the cartilage and muscle for postnatal individuals (P0, 14, 21, 30, 45, and adult), the diffusible iodine-based contrast-enhanced CT technique [55] was conducted. This method has been shown to effectively allow us to non-destructively investigate the morphology of bat soft tissues in detail [38, 39, 90–93]. Specimens were stained with 1% iodine in ethanol for at least 48 h and up to 14 days. The morphology of each hyolaryngeal component was manually segmented from the aligned serial tissue sections (Fig. 13) and micro-CT images (Fig. 14) in Amira 5.3 Table 4.

**Table 4:** Summary of measurements in each specimen in this study

Species	ID	Stage	GM^3^ (mm^3^)	First harmonic (kHz)	Second harmonic (kHz)	Cricoid cartilage volume (mm^3^)	Thyroid cartilage volume (mm^3^)	Arytenoid cartilage volume (mm^3^)	Lateral tracheal chamber volume (mm^3^)
*Rhinolophus cornutus*	JP21-045	Postnatal day 0	572.26			0.80	0.18	0.11	
*Rhinolophus cornutus*	JP21-046	Postnatal day 0	520.08			0.62	0.20	0.11	
*Rhinolophus cornutus*	JP21-047	Postnatal day 0	586.54			0.86	0.27	0.17	
*Rhinolophus cornutus*	JP21-073	Postnatal day 14	623.39	49.99	99.98	0.85	0.31	0.19	0.21
*Rhinolophus cornutus*	JP21-074	Postnatal day 14	645.35	49.58	99.20	0.93	0.37	0.22	0.27
*Rhinolophus cornutus*	JP21-075	Postnatal day 14	595.82	49.90	99.93	0.86	0.34	0.23	0.19
*Rhinolophus cornutus*	JP21-083	Postnatal day 21	644.23	50.67	101.23	0.87	0.35	0.23	0.32
*Rhinolophus cornutus*	JP21-097	Postnatal day 30	681.38	51.28	102.67	0.99	0.35	0.29	0.32
*Rhinolophus cornutus*	JP21-100	Postnatal day 30	681.38	50.89	101.83	1.01	0.41	0.27	0.31
*Rhinolophus cornutus*	JP21-101	Postnatal day 30	671.22	50.92	101.92	0.95	0.36	0.24	0.32
*Rhinolophus cornutus*	JP21-104	Postnatal day 45	701.12	51.69	103.37	1.06	0.38	0.31	0.36
*Rhinolophus cornutus*	JP21-103	Adult		54.14	108.25				
*Rhinolophus cornutus*	NHKM2937-	Adult	704.82			1.37	0.53	0.34	0.57
*Mus musculus*	JP19-001	E17.5	222.02						
*Mus musculus*	NT22-005	Postnatal day 0	288.58						
*Mus musculus*	NT22-007	Postnatal day 7	712.25						
*Mus musculus*	NT22-008	Postnatal day 14	1323.15				0.04		
*Mus musculus*	NT22-010	Adult	1748.84			0.38	0.73		

### Histology

Serial tissue sections were prepared to reconstruct the detailed morphology that is still difficult to visualize by the diffusible iodine-based contrast-enhanced CT. Three stages of bats (CS17, CS20.5, CS22) and mice (E14.5, E16.5, E18.5) were dehydrated in ethanol series and embedded in paraffin wax. Sections were cut at 6 μm for the CS17 bat and E14.5 mouse, and 7 μm for the other stages. For immunohistochemistry (IHC) staining of CS22 of *R. malayanus* and E18.5 of mice, mouse anti-acetylated tubulin (mouse monoclonal anti-acetylated tubulin, no. T7451; Sigma-Aldrich Japan, Tokyo) was used to visualize the innervations. For CS17 of *R. pusillus*, anti-Sox9 (rabbit polyclonal antibody, no. AB5535; Sigma-Aldrich Japan, Tokyo) was used to visualize the chondrifications. The immunoreaction was visualized by the secondary antibody conjugated with the anti-mouse IgG-Biotin antibody. The sections were then stained with Alcian blue and hematoxylin to identify the cartilages and muscles following the standard staining protocols. The obtained serial sections were aligned and then each targeted organ was manually segmented using Amira 5.3 (Visage Imaging GmbH).

### Measurements

To examine the allometric growth of the mineralized components against overall skull size in horseshoe bats, the skull size and volume of the mineralized laryngeal cartilages (cricoid, arytenoid, thyroid cartilages, lateral, and dorsal tracheal chambers) were measured using the Surface Area Tool and 3D Length Tool in Amira 5.3. As the index of overall skull size [94], the geometric mean (GM) was calculated from the skull length (SL), skull height (SH), and skull width (SW) as

$${{\text{GM}}}^{3}={\text{SL}}*{\text{SH}}*{\text{SW}}.$$

The cubed GM (GM^3^) was used as the index of the skull size in our allometric analyses. Skull length (SL) was measured as the distance from the rostral tip of the premaxilla to the ventral end of the interparietal in the sagittal plane. Skull height (SH) was measured as the distance from the midpoint of the cranial tip of the left and right parietals to the rostral tip of the basioccipital. Skull width (SW) was taken as the distance of the bilateral tip of the squamosal.

### Bioacoustics

Echolocation pulses of P0 (*n* = 3), P14 (*n* = 3), P21 (*n* = 1), P30 (*n* = 2), P45 (*n* = 1), and adult (*n* = 1) individuals were recorded, and sonograms were obtained during the field sampling using Echo Meter Touch 2 Pro (Wildlife Acoustics Inc., Concord, MA). The mean of the sound frequency of the first harmonic (CF_1_) and second harmonic (CF_2_) pulse of the constant frequency (CF) was taken as the index for the capability of the biosonar pulse emission using Kaleidoscope Pro Analysis Software (Wildlife Acoustics Inc., Concord, MA).

### Ossification heterochrony

To examine the temporal specificity of the hyolaryngeal ossification in horseshoe bats, the relative ossification timing of the hyolaryngeal components (arytenoid cartilage, basihyal, ceratohyal, corniculate cartilage, cricoid cartilage, epihyal, stylohyal, thyrohyal, thyroid cartilage, dorsal and lateral tracheal chambers) was estimated. The ossification sequence of 31 craniocervical elements (alisphenoid, arytenoid cartilage, basihyal, basioccipital, basisphenoid, ceratohyal, corniculate cartilage, cricoid cartilage, dentary, dorsal and lateral tracheal chambers, ectotympanic, epihyal, exoccipital, frontal, goniale, jugal, lacrimal, maxilla, nasal, orbitosphenoid, palatine, parietal, petrosal, premaxilla, presphenoid, pterygoid, squamosal, stylohyal, supraoccipital, thyroid cartilage, thyrohyal, and vomer) was documented for *Rhinolophus* species and *M. musculus*. To examine the relative ossification timing of each bony component and allow interspecific comparison, the rank of each ossification event was scaled as

$$\frac{(r-1)}{{(r}_{{\text{max}}}-1)},$$

in which *r* is the absolute rank of each ossification event, and *r*_max_ is the total number of ranks for each species. The scaled relative values are distributed between 0 and 1. As the sequence resolution can affect the results in this approach, > 3 ranks were documented for each species following [95].

### Statistics

All statistical analyses were performed in PAST4. To examine the size allometry of the volume of the mineralized laryngeal cartilages, the reduced major axis (RMA) regression analysis was conducted. To rescale the data and handle them on a common scale, the volume of the cricoid (CV), thyroid (TV), arytenoid (AV), and lateral tracheal chamber (CH) were log_10_-transformed and regressed against log_10_-transformed GM^3^. Furthermore, the regression line of the maximum frequency of the log10-transformed CF_1_ and CF_2_ components against log10-transformed GM^3^ was also obtained. Given that the RMA provides the best-estimated regression between every population from which the sample is selected when the error variance is unknown and is not affected by the correlation coefficient of samples, the RMA analysis is considered to be more appropriate than other regression models [96, 97].

To investigate the relationship between the pulse frequency and mineralization of the laryngeal cartilages, the generalized linear model (GLM) with identity link analyses was conducted. GLM analysis allows us to accommodate different types of response variables using the link function for various probability distributions and include the multiple independent variables in model, thus controlling for potential confounding factors that may influence the relationship between pulse peak frequency and mineralized volume of the laryngeal cartilages [98]. In the GLM analysis, the pulse frequency of CF_1_ and CF_2_ was set as the response variable, and the log-transformed volume of the mineralized cricoid cartilage, thyroid cartilage, arytenoid cartilage, lateral tracheal chamber, and dorsal tracheal chamber was as the explanatory variable. Since the pulse peak frequency is a continuous variable, it was assumed to follow a normal distribution. The volume of each mineralized laryngeal cartilage was divided by the estimated skull volume to remove the effect of the skull size growth (relative cricoid cartilage volume: RCV, relative thyroid cartilage volume: RTV, relative arytenoid cartilage volume: RAV, and relative lateral tracheal chamber volume: RCHV).

### Supplementary Information

**Additional file 1.** The coding of the craniocervical ossification or calcification in all specimens of *Rhinolophus *sp. and *Mus musculus,* examined this study.

**Additional file 2.** The ossification or calcification sequence of the craniocervical elements in all specimens of *Rhinolophus* sp. and *Mus musculus,* examined this study.

**Additional file 3.** The relative timing of the ossification or calcification of the craniocervical elements in all specimens of *Rhinolophus* sp. and *Mus musculus, *examined this study.

## Data Availability

All data supporting the findings of this study are available from the corresponding authors.

## Abbreviations

ac: Arytenoid cartilage
ba: Basihyal
cc: Cricoid cartilage
ce: Ceratohyal
cln: Cranial laryngeal nerve
co: Corniculate cartilage
cp: Corniculate process of the arytenoid cartilage
ct: Cricothyroid muscle
dca: Dorsal cricoarytenoid muscle
dch: Dorsal tracheal chamber
eg: Epiglottis
ep: Epihyal
ft: Foramen thyrohyoideum
g: Glottis
hc: Hyoid complex
lca: Lateral cricoarytenoid muscle
lch: Lateral tracheal chamber
mw: Muscular wing of the cricoid cartilage
pt: Protuberance of the thyroid cartilage
rln: Recurrent laryngeal nerve
sc: Sagittal crest of the cricoid cartilage
st: Stylohyal
t: Trachea
ta: Thyroarytenoid muscle
tc: Thyroid cartilage
tcac: Thyroid caudal cornua
tcrc: Thyroid cranial cornua
th: Thyrohyal
thm: Thyrohyoid muscle
tr: Tracheal ring
vn: Vagus nerve
vp: Vocal process of the arytenoid cartilage
